# Sequential Ugi reaction/base-induced ring closing/IAAC protocol toward triazolobenzodiazepine-fused diketopiperazines and hydantoins

**DOI:** 10.3762/bjoc.14.49

**Published:** 2018-03-14

**Authors:** Robby Vroemans, Fante Bamba, Jonas Winters, Joice Thomas, Jeroen Jacobs, Luc Van Meervelt, Jubi John, Wim Dehaen

**Affiliations:** 1 Molecular Design and Synthesis, Department of Chemistry, KU Leuven, Celestijnenlaan 200F, 3001 Leuven, Belgium; 2 Laboratoire de Chimie Organique Structurale, UFR Sciences des Structures de la Matiere et de Technologie, Universite Felix Houphouet-Boigny, Ivory Coast; 3 Biochemistry, Molecular and Structural Biology, Department of Chemistry, KU Leuven, Celestijnenlaan 200F, box 2404, 3001 Leuven, Belgium; 4 Organic Chemistry Section, CSIR-National Institute for Interdisciplinary Science and Technology (CSIR-NIIST), Thiruvananthapuram-19, India

**Keywords:** benzodiazepine, 2,5-diketopiperazine, hydantoin, intramolecular azide–alkyne cycloaddition, Ugi reaction

## Abstract

A practical three-step protocol for the assembly of triazolobenzodiazepine-fused diketopiperazines and hydantoins has been developed. The synthesis of these tetracyclic ring systems was initiated by an Ugi reaction, which brought together all necessary functionalities for further transformations. The Ugi adducts were then subjected to a base-induced ring closing and an intramolecular azide–alkyne cycloaddition reaction in succession to obtain highly fused benzodiazepine frameworks.

## Introduction

The versatile bioactivities of multiring-fused heterocyclic scaffolds continue to attract significant attention in developing new methods for their synthesis. A plethora of functionalized fused heterocycles can be easily accessed by designing sequential organic transformations incorporating multicomponent reactions [1–2] combined with secondary transformations [3–11]. Over the past decade, several groups of chemists interested in synthetic chemistry have efficiently utilized the strategy of linking multicomponent reactions with intramolecular azide–alkyne cycloaddition (IAAC) for the generation of triazole-fused heterocycles [12–23]. In this report we disclose our results on the development of a sequential synthetic approach involving Ugi 4-component reaction (4-CR) and two ring-closing steps toward triazolobenzodiazepine-fused diketopiperazines and hydantoins.

**Figure 1:**
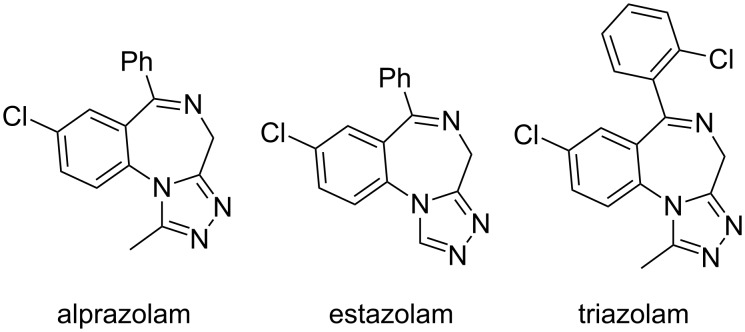
Triazolobenzodiazepine drugs.

Benzodiazepine derivatives [24–25] form an important class of ‘psychoactive drugs’ which is being extensively used in the treatment of anxiety, insomnia, agitation, seizures, muscle spasms, alcohol withdrawal, etc*.* [26–34]. In addition, these azaheterocycles also exhibit anti-inflammatory, antitumor, antiparasitic and anxiolytic activities [35–42]. In particular, 1,4-benzodiazepine derivatives are proposed to serve as a structural mimic of peptide β-turns [43–45] and are also known to bind to a number of biological targets [46–48]. Interestingly, the fusion of a triazole ring to a 1,4-benzodiazepine core has resulted in an increase in the biological activity as evident from the different drugs with triazolobenzodiazepine structure such as alprazolam, etizolam, triazolam, etc*.*, available for the treatment of CNS related ailments (Figure 1) [49–55].

**Scheme 1:**
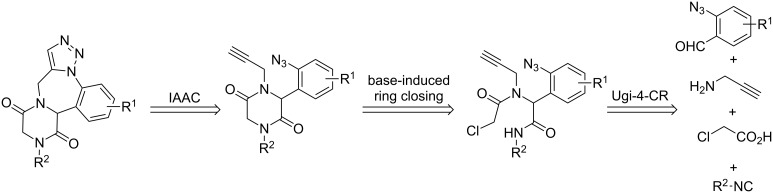
Retrosynthetic analysis towards 2,5-diketopiperazine fused triazolobenzodiazepine.

Another set of heterocycles are 2,5-diketopiperazines, which form the core structure of many natural products and have recently been thoroughly studied for their wide spectrum of biological activities [56–58]. Owing to our interest in the chemistry of 1,2,3-triazoles [59–72] and the interesting biological activities of benzodiazepines and 2,5-diketopiperazines, we were motivated to develop a facile route towards multiring-fused derivatives incorporating all these heterocyclic moieties. We hypothesized that 2,5-diketopiperazine-fused triazolobenzodiazepine could be obtained by proper functionalization of building blocks starting with an Ugi reaction; followed by two ring-closing steps, one being a base-induced heterocycle formation and the other an IAAC. Unlike some of the reported strategies using Cu-catalyzed IAAC [13,15], we postulated that the intramolecular dipolar cycloaddition would proceed under thermal conditions (metal-free) due to the close proximity of the azide and similar functionalities in the intermediate. The functionalities required for the secondary transformations like the leaving group (-Cl) for nucleophilic substitution, alkyne and azide for IAAC can be assembled via the Ugi reaction (Scheme 1).

## Results and Discussion

**Scheme 2:**
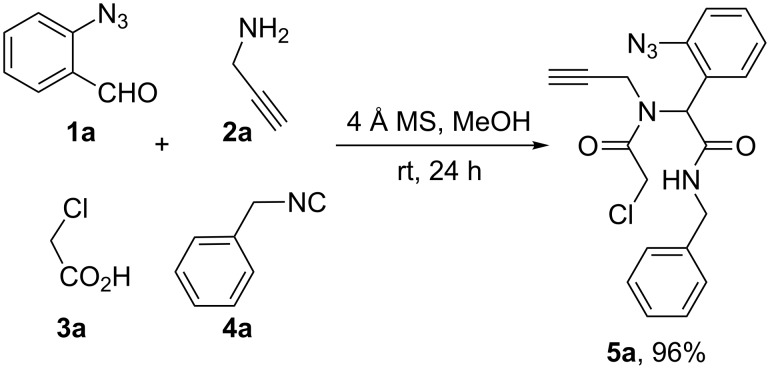
Ugi 4-CR reaction.

We initiated our studies with the Ugi 4-component reaction of *o*-azidobenzaldehyde (**1a**), propargylamine (**2a**), 2-chloroacetic acid (**3a**) and benzyl isocyanide (**4a**, Scheme 2). Initially, the condensation of the aldehyde and the amine was effected in MeOH in the presence of 4 Å MS. This was followed by the sequential addition of chloroacetic acid and the isocyanide which, after 24 hours at room temperature, afforded the expected Ugi adduct **5a** in 96% yield.

**Scheme 3:**
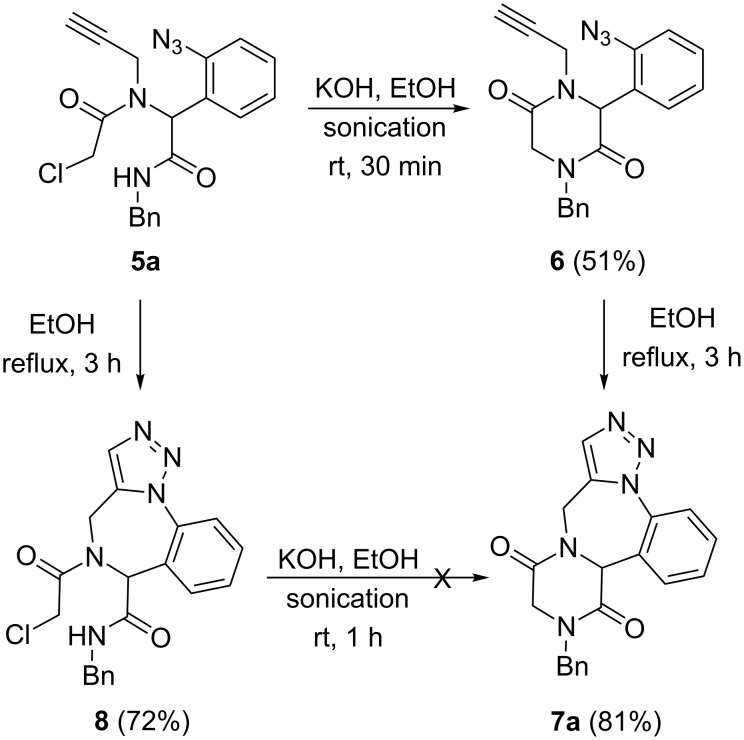
Synthesis of diketopiperazine-fused triazolobenzodiazepine **7a**.

The synthesis of fused heterocycles from the Ugi adduct **5a** was attempted via two routes (Scheme 3). The first route involved the base-induced cyclisation of **5a** at the amide end by treatment with KOH from which the diketopiperazine moiety **6** was obtained in 51% yield. Compound **6** bearing azide and alkyne functionalities was subjected to intramolecular azide–alkyne cycloaddition (IAAC) in EtOH at reflux conditions which afforded the target diketopiperazine-fused triazolobenzodiazepine **7a** in 81% yield. The secondary path commenced with the intramolecular azide–alkyne cycloaddition of **5a** which furnished the triazole-fused benzodiazepine **8** in 72% yield after 3 hours. The triazolobenzodiazepine **8** was then subjected to base-induced cyclisation with KOH which failed to afford the expected tetracyclic compound **7a**. We believe that conformational restrictions prevent the side chains on the benzodiazepine bearing the amide and halogen to be in close proximity, which is needed for the reaction to proceed.

**Figure 2:**
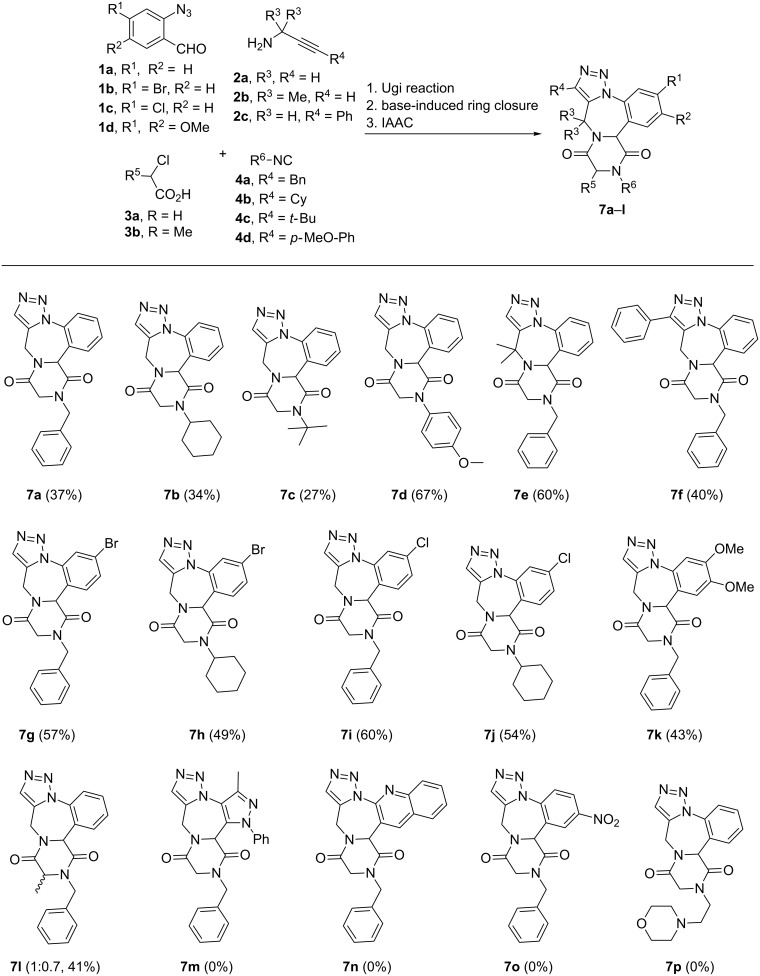
Generality in the synthesis of diketopiperazine-fused triazolobenzodiazepine **7**. Reaction conditions: 1. 2-azidobenzaldehyde **1** (1.85 mmol), propargylamine **2** (1.85 mmol), chloroacetic acid **3** (1.85 mmol), benzyl isocyanide **4** (1.85 mmol), 4 Å MS (100 mg), EtOH (7 mL), 20 h, rt; 2. Ugi adduct **5** (0.26 mmol), KOH (0.28 mmol), ethanol (1.5 mL), sonication for 30 minutes, rt; 3. neutralization with 1 M HCl, EtOH (20 mL) reflux, 3 h. Overall yields for 3 steps are given for all the examples.

The generality of the developed fused-heterocycle synthesis was studied with substituted azidoaldehyde, isocyanides and chloroacetic acid (Figure 2, see Supporting Information File 1 for full experimental data). In all the cases, the Ugi adduct precipitated from the reaction mixture within 24 hours, which was filtered, washed, dried and used as such without further purification. For all the examples, the ring-closing reactions were done in one-pot; which involved neutralization after base-induced diketopiperazine synthesis and dilution with EtOH before the IAAC step. Our first attempt was to study the effect of different substituents on the isocyanide (**4a**–**d**) moiety; from which we observed a slight decrease in the yield of the final product with increase in steric bulk of the substituent. Hence, an overall yield (over 3 steps) of 37% was obtained for benzyl-substituted polycyclic compound **7a**, 34% for **7b** (cyclohexyl) and 27% for **7c** (*tert*-butyl). *p*-Methoxyphenyl-substituted compound **7d** was obtained in a good overall yield of 67%. Next, we investigated the effect of the substitution pattern on the propargylamine **2**. For this, we chose easily available propargylamines **2b** and **2c** which gave moderate to good yields (60 and 40%, respectively) of diketopiperazine-fused triazolobenzodiazepine **7e** and **7f**. Furthermore, halogen substituents were introduced on polyfused heterocycles **7** because these were shown to often have a positive effect on the bioactivity (see compounds in Figure 1). Starting with 2-azido-4-bromobenzaldehyde (**1b**), we were able to prepare two brominated tetracyclic derivatives **7g** and **7h**. In the same way, we were also successful in synthesizing chloro derivatives **7i** and **7j** from 2-azido-4-chlorobenzaldehyde (**1c**). The multiring-fused heterocycle synthesis was also tried with an azidobenzaldehyde bearing electron-donating substituents; the reaction with 2-azido-4,5-dimethoxybenzaldehyde (**1d**) yielded the product **7k** in lesser yield (43%). Finally, an additional stereocenter was introduced in the diketopiperazine ring by carrying out the Ugi reaction with 2-chloropropanoic acid (**3b**). After the sequential ring-closing steps the expected diketopiperazine-fused triazolobenzodiazepine **7l** was obtained in 41% yield as a 1:0.7 inseparable mixture of diastereomers. Various attempts were made with other azidobenzaldehydes (5-azido-3-methyl-1-phenyl-1*H*-pyrazole-4-carbaldehyde, 2-azidoquinoline-3-carbaldehyde and 2-azido-5-nitrobenzaldehyde) and isocyanides (2-morpholinoethyl isocyanide) towards the synthesis of diketopiperazine-fused triazolobenzodiazepine **7m**–**p**. All of the mentioned substrates gave their respective Ugi adducts but the latter decomposed under basic treatment (in the case of 2-azidoquinoline-3-carbaldehyde, 2-azido-5-nitrobenzaldehyde, 2-morpholinoethyl isocyanide) or decomposed under refluxing conditions (in the case of 5-azido-3-methyl-1-phenyl-1*H*-pyrazole-4-carbaldehyde).

**Scheme 4:**
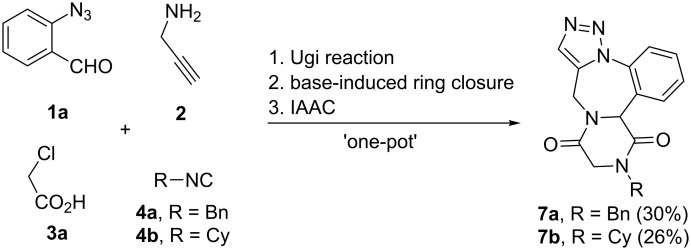
‘One-pot’ synthesis of diketopiperazine-fused triazolobenzodiazepines **7a** and **7b**.

The success in the synthesis of diketopiperazine-fused triazolobenzodiazepine prompted us to examine the possibility of making the sequential synthetic route in ‘one-pot’ starting from the Ugi 4-CR (Scheme 4). Thus we initiated the reaction sequence from the Ugi reaction in EtOH, after completion of which, the ring-closing steps (base induced and IAAC) were performed in succession. Unfortunately, the final products were obtained in slightly lower yields; 30% for **7a** and 26% for **7b**, respectively.

**Scheme 5:**
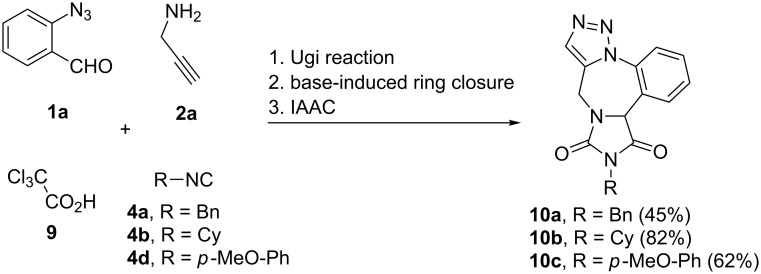
Synthesis of hydantoin-fused triazolobenzodiazepine **10**. Reaction conditions: 1. 2-azidobenzaldehyde **1** (1.85 mmol), propargylamine (**2a**, 1.85 mmol), trichloroacetic acid (**9**, 1.85 mmol), benzyl isocyanide **4** (1.85 mmol), 4 Å MS (100 mg), EtOH (7 mL), 20 h, rt; 2. Ugi adduct **5** (0.26 mmol), NaOEt (0.28 mmol), ethanol (1.5 mL), 30 min, rt; 3. neutralization with 1 M HCl, EtOH (20 mL) reflux, 3 h. Overall yields for 3 steps are given for all the examples.

Hydantoins belong to a unique class of heterocycles which can be found in a number of pharmaceuticals, cosmetics, insecticides, etc*.* [73–75]. These cyclic ureides are well known for their anticonvulsant activity [76–78]. With the assumption that these molecules when fused to a benzodiazepine core would enhance the anticonvulsant activity, we undertook the synthesis of hydantoin-fused triazolobenzodiazepines starting from the Ugi reaction of *o*-azidobenzaldehyde (**1a**), propargylamine (**2a**), trichloroacetic acid (**9**) and benzyl isocyanide (**4a**, Scheme 5). The Ugi adduct was subjected to base-induced ring closing with NaOEt in EtOH which furnished the hydantoin intermediates; which on subsequent neutralization–dilution–IAAC afforded the expected hydantoin-fused benzodiazepine derivative **10a** in 45% overall yield. In a similar way the cyclohexyl and *p*-methoxyphenyl-substituted compounds **10b** and **10c** were obtained in 82% and 62% overall yield, respectively.

**Figure 3:**
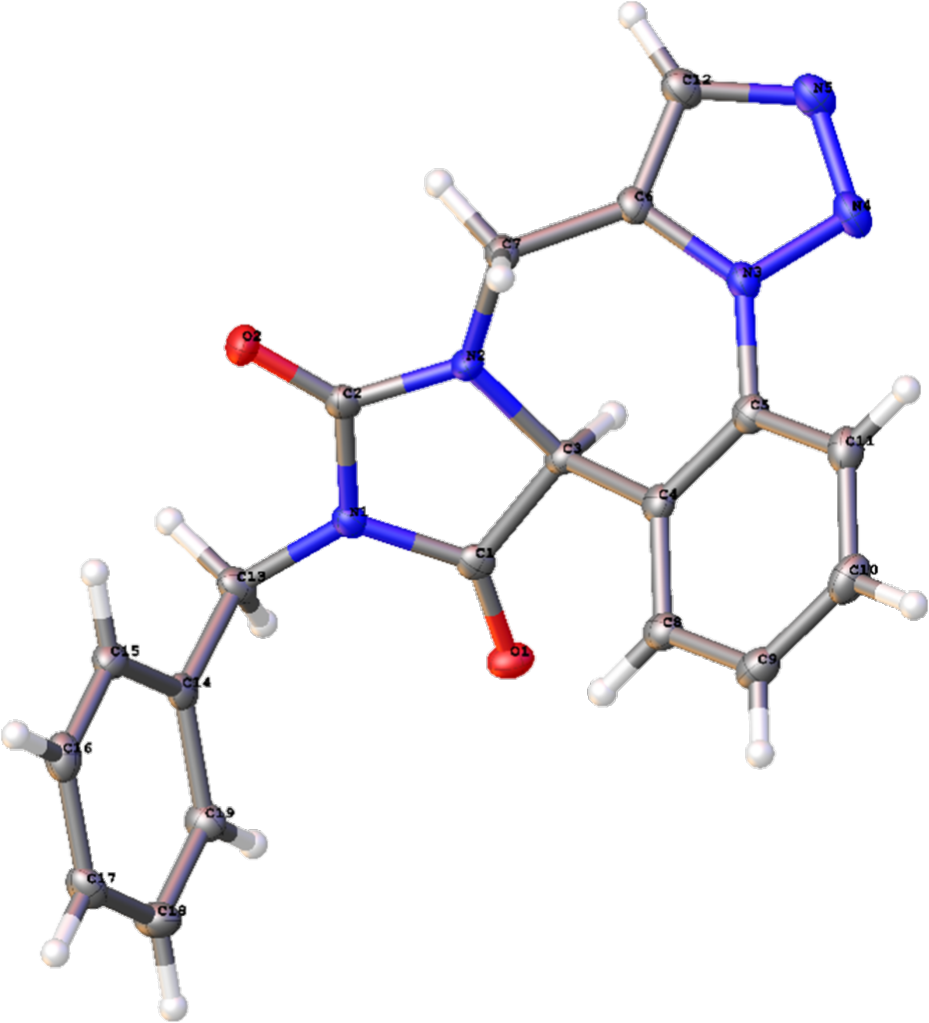
X-ray crystal structure of hydantoin-fused triazolobenzodiazepine **10a**. (Displacement ellipsoids are drawn at the 30% probability level.)

The structure of hydantoin-fused triazolobenzodiazepine was confirmed by a X-ray crystal structure determination of **10a** (Figure 3).

**Scheme 6:**
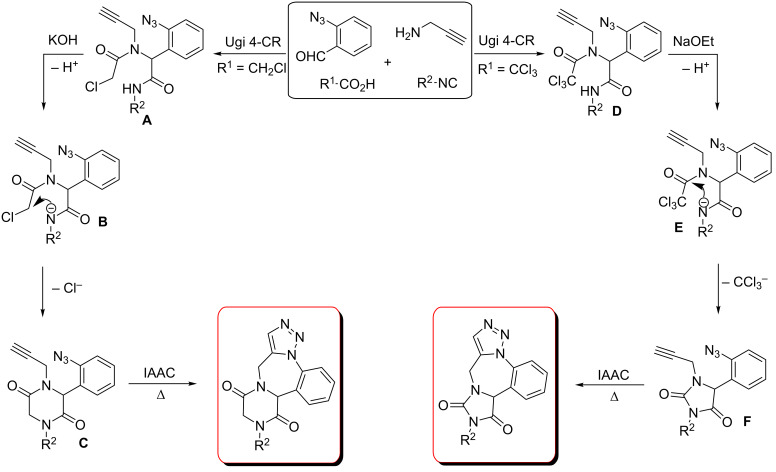
Mechanism of formation of diketopiperazine and hydantoin-fused triazolobenzodiazepines.

The three-step synthesis of heterocycle-fused triazolobenzodiazepines involves the well-known Ugi 4-CR as the first step and the IAAC as the last, the mechanisms of which are well established (Scheme 6). The second stage is the base-induced ring closing of the Ugi adduct **A** and **D** to the respective heterocycles. In the case of Ugi adduct **A**, the base (KOH) abstracts the amide hydrogen which attacks the methylene carbon (expelling a chloride ion) and thereby furnishing the 2,5-diketopiperazine ring **C**. On the other hand, in the case of Ugi adduct **D**, a base induced intramolecular nucleophilic attack of the amide N-atom to the carbonyl carbon occurs (in intermediate **E**) and the hydantoin ring **F** forms by the elimination of the trichloromethyl anion.

## Conclusion

In conclusion, we have developed a sequential synthetic strategy to access diketopiperazine-fused triazolobenzodiazepines from simple starting materials. It is noteworthy to mention that halogenated (chloro and bromo) benzodiazepines could be synthesized following the developed protocol which might show analogues biological activity as that of halogenated triazolobenzodiazepine drugs available in market. We could also extend the strategy for the synthesis of hydantoin-fused triazolobenzodiazepines, other valuable multiring-fused heterocycles which might also exhibit potent bioactivities. The synthesized compounds are currently being evaluated for their biological properties, the results of which will be reported in due course.

## Supporting Information

The Supporting Information features further experimental details, copies of ^1^H and ^13^C NMR spectra of compounds **5a**, **6**, **8**, diketopiperazine-fused triazolobenzodiazepines **7** and hydantoin-fused triazolobenzodiazepines **10**, and X-ray crystal structure details of **10a**.

File 1 Experimental part.
