# Supplementary material for: Sequential Ugi reaction/base-induced ring closing/IAAC protocol toward triazolobenzodiazepine-fused diketopiperazines and hydantoins
Source: Beilstein J Org Chem. 2018 Mar 14;14:626–33. doi: 10.3762/bjoc.14.49 (PMC5870159; doi:10.3762/bjoc.14.49)
Supplement: File 1 — Experimental part. [file Beilstein_J_Org_Chem-14-626-s001.pdf]

Supporting information

for

Sequential Ugi reaction/base-induced ring closing/IAAC protocol toward triazolobenzodiazepine-fused diketopiperazines and hydantoins

Robby Vroemans[1‡], Fante Bamba[1,2‡], Jonas Winters[1], Joice Thomas[1], Jeroen Jacobs[3], Luc Van Meervelt[3], Jubi John[1,4*] and Wim Dehaen[1*]

Address: [1]Molecular Design and Synthesis, Department of Chemistry, KU Leuven, Celestijnenlaan 200F, 3001 Leuven, Belgium, [2]Laboratoire de Chimie Organique Structurale, UFR Sciences des Structures de la Matiere et de Technologie, Universite Felix Houphouet-Boigny, Ivory Coast, [3]Biochemistry, Molecular and Structural Biology, Department of Chemistry, KU Leuven, Celestijnenlaan 200F, box 2404, 3001 Leuven, Belgium and [4]Organic Chemistry Section, CSIR-National Institute for Interdisciplinary Science and Technology (CSIR-NIIST), Thiruvananthapuram-19, India

Email: Wim Dehaen - wim.dehaen@chem.kuleuven.be; Jubi John -jubijohn@niist.res.in

‡ Equally contributing authors

* Corresponding author

Experimental part

General

NMR spectra were acquired on commercial instruments (Bruker Avance 300 MHz, Bruker AMX 400 MHz or Bruker Avance II$^+$ 600 MHz) and chemical shifts (δ) are reported in parts per million (ppm) referenced to tetramethylsilane (^{1}H), or the internal (NMR) solvent signal (^{13}C). Mass spectra were run using a HP5989A apparatus (EI, 70 eV ionisation energy) with Apollo 300 data system, a Micromass Quattro II apparatus (ESI) with MASSLYNX data system or a Thermo Finnigan LCQ Advantage apparatus (ESI). Exact mass measurements were acquired on a Kratos MS50TC instrument (performed in the EI mode at a resolution of 10000. Melting points (not corrected) were determined using a Reichert Thermovar apparatus. For column chromatography 70–230 mesh silica 60 (E. M. Merck) was used as the stationary phase. Chemicals received from commercial sources were used without further purification. Reaction solvents were used as received from commercial sources. 2-Azidobenzaldehydes **1a**–**d** [1-4] and 3-phenylprop-2-yn-1-amine (**2c**) [5] were prepared according to literature reports.

Typical procedure for Ugi reaction: Synthesis of 2-(2-azidophenyl)-*N*-benzyl-2-(2-chloro-*N*-(prop-2-yn-1-yl)acetamido)acetamide (**5a**): To an oven-dried screw-capped reaction vial equipped with a magnetic stirring bar 2-azidobenzaldehyde (**1a**, 272 mg, 1.85 mmol), propargylamine (**2a**, 71 µL, 1.85 mmol), 4 Å molecular sieves (100 mg) and ethanol (10 mL) were added. The mixture was stirred at room temperature under argon for one hour. Chloroacetic acid (**3a**, 173 mg, 1.85 mmol) and benzyl isocyanide (**4a**, 225 µL, 1.85 mmol) were then added and stirred for an additional 23 hours at room temperature under argon atmosphere. The suspension was filtered and the product washed with pentane (100 mL). The Ugi adduct **5a** was obtained as an off-white solid (703 mg, 96% yield). Mp: 193-195 °C; (^{1}H NMR, 300 MHz, $CDCl_3$)

δ: 2.05 (s, 1H), 4.14 (s, 2H), 4.32 (s, 2H), 4.48 (s, 2H), 6.22 (s, 1H), 7.18 (d, *J* = 7.8 Hz, 2H), 7.30 (m, 5H), 7.44 (t, *J* = 7.1 Hz, 2H);(^{1}H NMR, 600 MHz, CD_3CN, 7:3 mixture of diastereomers. The Ugi adduct bears two amide moieties and hence the ^{1}H and ^{13}C NMR spectra of these compounds are broadened due to the presence of rotamers [16]) δ: 1.96 (s, 1H), 3.86 (d, *J* = 15.1 Hz, 1H), 4.07-3.85 (m, 2 H), 4.22 (d, *J* = 14.6 Hz, 0.7H), 4.49-4.42 (m, 2H), 5.12 (d, *J* = 14.5 Hz, 0.7H), 5.49 (d, *J* = 15.0 Hz, 0.3H), 5.65 (s, 0.3H), 6.22 (s, 0.7H), 6.81 (s, 1H), 6.95 (d, *J* = 7.0 Hz, 2H), 7.27-7.19 (m, 3H), 7.71-7.55 (m, 3H), 7.88 (d, *J* = 7.7 Hz, 1H); (^{13}C NMR, 151 MHz, CD_3CN, diastereomeric mixture and amide rotameric effect) δ: 37.5, 39.2, 43.1, 43.5, 43.7, 43.9, 61.9, 64.1, 124.3, 124.5, 127.9, 128.1, 128.2, 128.5, 128.8, 129.2, 129.3, 130.6, 130.7, 131.7, 132.0, 132.3, 132.7, 134.1, 134.2, 134.4, 134.7, 136.1, 136.2, 139.4, 139.6, 167.3, 167.5, 168.3, 168.4; Exact mass (HRMS**,** ESI) calculated for $C_{20}H_{18}ClN_5O_2$ $(M+H)^+$: 396.1221, found 396.1210.

Typical procedure for base induced cyclisation of Ugi adducts towards 2,5-diketopiperazine: Synthesis of 3-(2-azidophenyl)-1-benzyl-4-(prop-2-yn-1-yl)piperazine-2,5-dione (**6**): Ugi adduct **5a** (100 mg, 0.26 mmol) was added to a solution of KOH (16 mg, 0.28 mmol) in ethanol (1.5 mL). The reaction mixture was ultrasonicated for 30 minutes and subsequently neutralized with 1 N HCl. The reaction mixture was concentrated under reduced pressure and water was added which was then worked up with 10 mL of EtOAc three times. The combined organic layer was dried over anhydrous $MgSO_4$ and concentrated under reduced pressure. The crude reaction was purified by silica gel column chromatography (EtOAc/heptane = 30:70) affording diketopiperazine **6** (46 mg, 51% yield) as a white solid. Mp: 108-110 °C; (^{1}H NMR, 300 MHz, $CDCl_3$) δ: 2.28 (t, *J* = 2.5 Hz, 1H), 3.12 (dd, *J* = 17.5, 2.4 Hz, 1H), 3.97 (d, *J* = 17.7 Hz, 1H), 4.13 (d, *J* = 17.7 Hz, 1H), 4.27

(d, J = 14.3 Hz, 1H), 4.87 (dd, J = 17.5, 2.6 Hz, 1H), 5.03 (d, J = 14.3 Hz, 1H), 5.43 (s, 1H), 7.21-7.16 (m, 2H), 7.34-7.31 (m, 4H), 7.48-7.37 (m, 3H); (^{13}C NMR, 75 MHz, $CDCl_3$) δ: 32.5, 49.0, 49.4, 50.1, 58.8, 58.8, 60.7, 60.7, 73.6, 73.6, 119.0, 125.3, 126.5, 128.8, 128.9, 129.0, 129.1, 129.2, 130.6, 130.9, 132.4, 132.6, 133.7, 134.1, 134.8, 134.8, 135.9, 138.0, 162.5, 162.9, 163.1, 163.7; (HRMS**,** ESI) calculated for $C_{20}H_{17}N_5O_2$ $(M+H)^+$: 360.1455, found 360.1449.

Typical procedure for intramolecular azide–alkyne cycloaddition of 2,5-diketopiperazine: Synthesis of 13-benzyl-12,13-dihydrobenzo[*f*]pyrazino[1,2-*d*][1,2,3]triazolo[1,5-*a*][1,4]diazepine-11,14(9*H*,14a*H*)-dione **7a**: Diketopiperazine **6** (50 mg, 0.14 mmol) was refluxed in EtOH (10 mL) for three hours. Upon completion, the reaction mixture was concentrated under reduced pressure and the residue on silica gel chromatography yielded the triazolobenzodiazepine **7a** as a pale solid (41 mg, 81%).Mp: 166-168 °C; (^{1}H NMR, 600 MHz, $CDCl_3$) δ: 4.04 (s, 2H), 4.40 (d, 1H, J = 16.2 Hz), 4.74 (d, 1H, J = 14.4 Hz), 4.83 (d, 1H, J = 13.8 Hz), 5.08 (s, 1H), 5.09 (d, 1H, J = 15.0 Hz), 7.21 (d, 1H, J = 7.8 Hz), 7.39-7.45 (m, 5H), 7.49 (t, 1H, J = 7.2 Hz), 7.63 (t, 1H, J = 7.8 Hz), 7.84 (s, 1H) 8.00 (d, 1H, J = 7.8 Hz); (^{13}C NMR, 150 MHz, $CDCl_3$) δ: 38.7, 48.9, 50.0, 58.7, 124.1, 126.4, 128.7, 128.9, 129.1, 129.2, 130.0, 130.6, 130.7, 134.0, 134.7, 135.8, 162.4, 163.0; Exact mass (HRMS, ESI) calculated for $C_{20}H_{17}N_5O_2$ $(M+H)^+$: 360.1461, found: 360.1460.

***N*-Benzyl-5-(2-chloroacetyl)-5,6-dihydro-4H-benzo[*f*][1,2,3]triazolo[1,5-*a*][1,4]diazepine-6-carboxamide** (**8**)**:** Ugi adduct **5a** (50 mg, 0.13 mmol) was dissolved in 1,4-dioxane (5 mL) and refluxed for three hours. The reaction mixture was concentrated under reduced pressure and the product was purified by column chromatography (EtOAc/heptane = 50:50) affording **8** (36 mg, 72% yield) as an off-white solid. Mp: 182-184 ˚C; (^{1}H NMR,600 MHz, MeOD, 7:3 diastereomeric mixture

and amide rotameric effect) δ: 4.05 (m, 2H), 4.34 (m, 1H), 4.57 (m, 2H), 5.31 (d, *J* = 14.8 Hz, 0.7H), 5.56 (d, *J* = 15.6 Hz, 0.3H), 5.92 (s, 0.3H), 6.24 (s, 0.7H), 6.96 (m, 2H), 7.28-7.14 (m, 3H), 7.65-7.57 (m, 1H), 7.71 (d, *J* = 6.7 Hz, 2H), 7.80 (s, 1H), 7.91 (d, *J* = 7.8 Hz, 1H); (^{13}C NMR,75 MHz, DMSO-D_6) δ: 30.4, 34.4, 42.2, 43.4, 60.6, 60.9, 123.2, 126.7, 126.8, 127.2, 128.1, 128.4, 129.4, 129.5, 130.5, 130.6, 131.6, 133.1, 133.4, 133.5, 134.0, 134.1, 134.7, 138.7, 138.8, 138.9, 139.2, 166.1, 166.2, 167.4, 167.5; (HRMS,ESI) calculated for $C_{20}H_{18}ClN_5O_2$ $(M+H)^+$: 396.1221, found 396.1217.

Typical procedure for the synthesis of 2,5-diketopiperazine derivatives 7a–l: To an oven-dried screw-capped reaction vial equipped with a magnetic stirring bar 2-azidobenzaldehyde **1a**–**d** (0.2 mmol), propargylamine **2a–c** (0.2 mmol), 4 Å molecular sieves (50 mg) and ethanol (2 mL) were added. The mixture was stirred at room temperature under argon for one hour. Chloroacetic acid **3a**,**b** (0.2 mmol) and isocyanide **4a**–**d** (0.2 mmol) were then added and stirred for an additional 23 hours at room temperature under argon atmosphere. The suspension was filtered and the product washed with pentane (100 mL). The Ugi adduct was dried and taken to the next step without further purification. The subsequent ring-closing steps were performed in one-pot starting with the base-induced cyclisation. The Ugi adduct was sonicated with 1.1 equiv of KOH in 2 mL of EtOH for 30 minutes. Upon completion, the reaction mixture was neutralized with 1 N HCl and diluted with 8 mL of EtOH. This mixture was refluxed for three hours after which the reaction mixture was concentrated under reduced pressure and the residue on silica gel chromatography (heptane/EtOAc mixtures as eluent) yielded the corresponding diketopiperazine-fused triazolobenzodiazepine.

13-Cyclohexyl-12,13-dihydrobenzo[*f*]pyrazino[1,2-*d*][1,2,3]triazolo[1,5-*a*][1,4] diazepine-11,14(9*H*,14a*H*)-dione (**7b**): Yield: 34% (24 mg); White solid - Mp: 248-250 °C; (^{1}H NMR, 600 MHz, $CDCl_3$) δ: 1.46-1.56 (m, 5H), 1.75-1.76 (m, 2H), 1.89-1.94 (m, 3H), 3.98 (d, 1H, *J* = 18.0 Hz), 4.09 (d, 1H, *J* = 17.4 Hz), 4.54 (d, 1H, *J* = 16.2 Hz), 4.58-4.62 (m, 1H), 5.03 (s, 1H), 5.06 (d, 1H, *J* = 16.2 Hz), 7.26 (d, 1H, *J* = 7.2 Hz), 7.52 (t, 1H, *J* = 7.8 Hz), 7.64 (t, 1H, *J* = 7.8 Hz), 7.84 (s, 1H) 8.03 (d, 1H, *J* = 7.8 Hz); (^{13}C NMR, 150 MHz, $CDCl_3$) δ: 25.2, 25.3, 25.5, 29.1, 30.1, 38.9, 44.7, 53.1, 59.0, 67.1, 124.2, 126.1, 129.2, 129.9, 130.7, 134.0, 135.9, 162.0, 163.7; Exact mass (HRMS, ESI) calculated for $C_{19}H_{21}N_5O_2$ $(M+H)^+$: 352.1773, found: 352.1780.

13-(*tert*-Butyl)-12,13-dihydrobenzo[*f*]pyrazino[1,2-*d*][1,2,3]triazolo[1,5-*a*][1,4] diazepine-11,14(9*H*,14a*H*)-dione (**7c**): Yield: 27% (18 mg); White solid - Mp: 212-214 °C; (^{1}H NMR, 300 MHz, $CDCl_3$) δ: 1.60 (s, 9H), 4.04 (d, 1H, *J* = 17.4 Hz), 4.25 (d, 1H, *J* = 17.4 Hz), 4.51 (d, 1H, *J* = 16.2 Hz), 4.93 (s, 1H), 5.00 (d, 1H, *J* = 16.2 Hz), 7.27-7.30 (merged doublet, 1H), 7.52 (t, 1H, *J* = 7.8 Hz), 7.62 (t, 1H, *J* = 7.8 Hz), 7.82 (s, 1H) 8.00 (d, 1H, *J* = 8.1 Hz); (^{13}C NMR, 75 MHz, $CDCl_3$) δ: 27.9, 38.6, 46.9, 59.1, 59.3, 124.2, 126.1, 129.7, 129.9, 130.5, 130.6, 133.9, 135.9, 163.2, 164.1; Exact mass (HRMS, ESI) calculated for $C_{17}H_{19}N_5O_2$ $(M+H)^+$: 326.1617, found: 326.1620.

13-(4-Methoxyphenyl)-12,13-dihydrobenzo[*f*]pyrazino[1,2-*d*][1,2,3]triazolo[1,5-*a*][1,4]diazepine-11,14(9*H*,14a*H*)-dione (7d): Yield: 67% (50 mg); White solid - Mp: 226-229 °C; (^{1}H NMR, 300 MHz, $CDCl_3$) δ: 3.85 (s, 3H), 4.35 (d, *J*=17.6, 1H), 4.54 (d, *J*=17.6, 1H), 4.64 (d, *J*=16.1, 1H), 5.11 (d, *J*=16.2, 1H), 5.20 (s, 1H), 7.04 – 6.97 (m, 2H), 7.36 – 7.29 (m, 2H), 7.42 (dd, *J*=7.7, 1.3, 1H), 7.55 (td, *J*=7.6, 1.3, 1H), 7.66 (td, *J*=7.8, 1.4, 1H), 7.87 (d, *J*=0.9, 1H), 8.05 (dd, *J*=8.0, 1.2, 1H); (^{13}C NMR, 75 MHz, $CDCl_3$) δ: 39.20, 53.03, 55.73, 59.50, 115.10, 124.43, 126.32, 126.76, 129.12,

130.10, 130.82, 130.99, 132.14, 134.15, 136.02, 159.26, 162.84, 163.50; Exact mass (HRMS, ESI) calculated for $C_{20}H_{17}N_5O_3$ $(M+H)^+$: 376.1404, found: 376.1401.

13-Benzyl-9,9-dimethyl-12,13-dihydrobenzo[*f*]pyrazino[1,2-*d*][1,2,3]triazolo[1,5-*a*][1,4]diazepine-11,14(9*H*,14a*H*)-dione (**7e**): Yield: 60% (47 mg); White solid - Mp: 230-232 °C; (^{1}H NMR, 300 MHz, $CDCl_3$) δ: 1.45 (s, 3H), 2.13 (s, 3H), 3.31 (d, *J*=14.4, 1H), 3.60 (d, *J*=14.4, 1H), 4.13 (qd, *J*=14.8, 5.8, 2H), 5.58 (s, 1H), 6.93 – 6.81 (m, 2H), 7.26 – 7.20 (m, 1H), 7.33 – 7.26 (m, 2H), 7.73 – 7.51 (m, 4H), 7.97 (dd, *J*=7.9, 0.9, 1H); (^{13}C NMR, 75 MHz, $CDCl_3$) δ: 25.74, 28.46, 44.16, 47.00, 54.16, 63.29, 125.64, 127.53, 127.88, 128.14, 128.17, 128.93, 129.37, 129.93, 129.96, 131.34, 136.34, 136.92, 163.93, 168.95; Exact mass (HRMS, ESI) calculated for $C_{22}H_{21}N_5O_2$ $(M+H)^+$: 388.1768, found: 388.1772.

13-Benzyl-8-phenyl-12,13-dihydrobenzo[*f*]pyrazino[1,2-*d*][1,2,3]triazolo[1,5-*a*][1,4]diazepine-11,14(9*H*,14a*H*)-dione (**7f**): Yield: 40% (35 mg); Off-white solid - Mp: 246-248 °C; (^{1}H NMR, 600 MHz, $CDCl_3$) δ: 4.09 – 4.00 (m, 2H), 4.45 (d, *J*=16.1, 1H), 4.74 (d, *J*=14.2, 1H), 4.80 (d, *J*=14.2, 1H), 5.17 (s, 1H), 5.32 (d, *J*=16.1, 1H), 7.23 (d, *J*=7.7, 1H), 7.47 – 7.36 (m, 6H), 7.53 – 7.48 (m, 3H), 7.67 – 7.62 (m, 1H), 7.73 – 7.69 (m, 2H), 8.03 (dd, *J*=7.9, 0.7, 1H); (^{13}C NMR, 151 MHz, $CDCl_3$) δ: 38.93, 49.12, 50.14, 58.95, 124.35, 126.60, 126.73, 128.07, 128.82, 129.06, 129.24, 129.36, 129.71, 130.18, 130.91, 134.80, 136.16, 146.78, 162.58, 163.24; Exact mass (HRMS, ESI) calculated for $C_{26}H_{21}N_5O_2$ $(M+H)^+$: 436.1768, found: 436.1766.

13-Benzyl-3-bromo-12,13-dihydrobenzo[*f*]pyrazino[1,2-*d*][1,2,3]triazolo[1,5-*a*][1,4] diazepine-11,14(9*H*,14a*H*)-dione (**7g**): Yield: 57% (50 mg); Pale solid - Mp: 83-85 °C; (^{1}H NMR, 300 MHz, $CDCl_3$) δ: 4.02 (s, 2H), 4.41 (d, 1H, *J* = 16.2 Hz), 4.66 (d, 1H, *J* = 14.1 Hz), 4.83 (d, 1H, *J* = 14.1 Hz), 5.03 (s, 1H), 5.09 (d, 1H, *J* = 16.2

Hz), 7.04 (d, 1H, *J* = 8.4 Hz), 7.40-7.42 (m, 5H), 7.57-7.61 (dd, 1H, J_1= 8.4 Hz, J_2= 1.8 Hz), 7.82 (s, 1H) 8.16 (d, 1H, *J* = 2.1 Hz); (^{13}C NMR, 150 MHz, $CDCl_3$) δ: 38.6, 48.9, 50.1, 58.4, 124.3, 127.1, 128.0, 128.8, 128.9, 129.2, 130.7, 133.0, 134.1, 134.6, 136.7, 162.0, 162.8; Exact mass (HRMS, ESI) calculated for $C_{20}H_{16}BrN_5O_2$ $(M+H)^+$: 438.0565, found: 438.0566.

3-Bromo-13-cyclohexyl-12,13-dihydrobenzo[*f*]pyrazino[1,2-*d*][1,2,3]triazolo[1,5-*a*][1,4]diazepine-11,14(9*H*,14a*H*)-dione (7h): Yield: 49% (42 mg); Pale solid - Mp: 135-137 °C; (^{1}H NMR, 300 MHz, $CDCl_3$) δ: 1.45-1.52 (m, 2H), 1.65-1.70 (m, 4H), 1.75-1.79 (m, 2H), 1.89-1.93 (m, 2H), 3.97 (d, 1H, *J* = 18.0 Hz), 4.11 (d, 1H, *J* = 18.0 Hz), 4.55-4.61 (m, 2H), 5.00 (s, 1H), 5.08 (d, 1H, *J* = 16.2 Hz), 7.13 (d, 1H, *J* = 8.1 Hz), 7.64-7.67 (dd, 1H, J_1= 8.1 Hz, J_2= 1.8 Hz), 7.85 (s, 1H) 8.23 (d, 1H, *J* = 2.1 Hz); (^{13}C NMR, 150 MHz, $CDCl_3$) δ: 25.2, 25.3, 25.5, 29.1, 30.1, 38.8, 44.7, 53.2, 58.7, 65.8, 124.2, 127.2, 127.7, 128.0, 130.9, 132.9, 134.1, 136.8, 161.6, 163.5; Exact mass (HRMS, ESI) calculated for $C_{19}H_{20}BrN_5O_2$ $(M+H)^+$: 430.0878, found: 430.0870.

13-Benzyl-3-chloro-12,13-dihydrobenzo[*f*]pyrazino[1,2-*d*][1,2,3]triazolo[1,5-*a*][1,4]diazepine-11,14(9*H*,14a*H*)-dione (7i): Yield: 60% (47 mg); White solid - Mp: 235-237 °C; (^{1}H NMR, 300 MHz, $CDCl_3$) δ: 4.04 (s, 2H), 4.41 (d, 1H, *J* = 16.2 Hz), 4.59 (d, 1H, *J* = 14.4 Hz), 4.95 (d, 1H, *J* = 14.1 Hz), 5.04 (s, 1H), 5.07 (Merged doublet, 1H), 7.14 (d, 1H, *J* = 1.8 Hz), 7.39-7.43 (m, 5H), 7.56-7.60 (dd, 1H, J_1= 8.7 Hz, J_2= 2.1 Hz), 7.83 (s, 1H) 7.93 (d, 1H, *J* = 9.0 Hz); (^{13}C NMR, 75 MHz, $CDCl_3$) δ: 38.7, 48.9, 50.2, 58.3, 125.3, 126.8, 128.8, 128.9, 129.3, 130.5, 130.6, 130.9, 134.1, 134.2, 134.6, 135.8, 161.8, 162.9; Exact mass (HRMS, ESI) calculated for $C_{20}H_{16}ClN_5O_2$ $(M+H)^+$: 394.1071, found: 394.1066.

3-Chloro-13-cyclohexyl-12,13-dihydrobenzo[*f*]pyrazino[1,2-*d*][1,2,3]triazolo[1,5-*a*][1,4]diazepine-11,14(9*H*,14a*H*)-dione (7j): Yield: 54% (42 mg); Pale solid - Mp: 115-117 ^{o}C; (^{1}H NMR, 300 MHz, $CDCl_3$) δ: 1.46-1.79 (m, 8H), 1.92-1.98 (m, 2H), 4.01 (d, 1H, J = 17.7 Hz), 4.12 (d, 1H, J = 18.0 Hz), 4.56 (d, 1H, J = 16.2 Hz), 4.61-4.63 (m, 1H), 5.02 (s, 1H), 5.09 (d, 1H, J = 17.1 Hz), 7.22 (d, 1H, J = 2.1 Hz), 7.61-7.64 (dd, 1H, J_1= 8.7 Hz, J_2= 2.1 Hz), 7.85 (s, 1H) 7.99 (d, 1H, J = 8.4 Hz); (^{13}C NMR, 150 MHz, $CDCl_3$) δ: 25.2, 25.3, 25.5, 29.1, 30.0, 38.9, 44.7, 53.3, 58.7, 125.4, 126.6, 130.6, 130.7, 130.8, 134.0, 134.4, 135.7, 161.3, 163.5; Exact mass (HRMS, ESI) calculated for $C_{19}H_{20}ClN_5O_2$ $(M+H)^+$: 386.1383, found: 386.1379.

13-Benzyl-2,3-dimethoxy-12,13-dihydrobenzo[*f*]pyrazino[1,2-*d*][1,2,3]triazolo[1,5-*a*][1,4]diazepine-11,14(9*H*,14a*H*)-dione (7k): Yield: 43% (36 mg); Pale solid - Mp: 163-165 ^{o}C; (^{1}H NMR, 300 MHz, $CDCl_3$) δ: 3.64 (s, 3H), 3.96 (s, 3H), 4.10 (d, 2H, J = 5.7 Hz), 4.21 (d, 1H, J = 14.1 Hz), 4.32 (d, 1H, J = 15.9 Hz), 5.02 (s, 1H), 5.07 (d, 1H, J = 15.9 Hz), 5.27 (d, 1H, J = 14.1 Hz), 6.43 (s, 1H), 7.38-7.49 (m, 6H), 7.81 (s, 1H); (^{13}C NMR, 75 MHz, $CDCl_3$) δ: 38.7, 49.5, 50.5, 56.1, 56.5, 58.5, 106.9, 108.1, 121.2, 128.7, 128.9, 129.1, 129.2, 130.5, 133.9, 135.1, 149.9, 150.4, 162.5, 162.9; Exact mass (HRMS, ESI) calculated for $C_{22}H_{21}N_5O_4$ $(M+H)^+$: 420.1671, found: 420.1681.

13-Benzyl-12-methyl-12,13-dihydrobenzo[*f*]pyrazino[1,2-*d*][1,2,3]triazolo[1,5-*a*][1,4]diazepine-11,14(9*H*,14a*H*)-dione (7l): Yield: 41% (31 mg); 1:0.7 Mixture of diastereomers; Pale solid - Mp: 180-182 ^{o}C; (^{1}H NMR, 300 MHz, $CDCl_3$) δ: 1.56 (d, 2.2H, J = 6.9 Hz), 1.65 (d, 3H, J = 6.9 Hz), 4.06-4.24 (m, 4.8H), 4.37 (d, 0.9H, J = 16.2 Hz), 5.06-5.32 (m, 4H), 5.47.5.58 (m, 1.8H), 7.28-7.31 (m, 2H), 7.38-7.47 (m, 9.6H), 7.51-7.70 (m, 4.7H), 7.85-7.86 (m, 1.7H), 7.92-7.95 (m, 1H), 8.04-8.07 (m, 0.8H); (^{13}C NMR, 75 MHz, $CDCl_3$) δ: 19.1, 19.2, 37.6, 39.2, 47.2, 47.4, 54.6, 54.7,

58.5, 58.8, 123.7, 124.0, 126.8, 128.3, 128.4, 128.5, 128.6, 128.9, 129.1, 129.4, 129.9, 130.0, 130.5, 130.7, 130.8, 130.8, 134.2, 135.0, 135.2, 135.8, 136.1, 162.2, 162.8, 165.8, 166.2; Exact mass (HRMS, ESI) calculated for $C_{21}H_{19}N_5O_2$ $(M+H)^+$: 374.1617, found: 374.1620.

2.5. Typical procedure for the synthesis of hydantoin fused triazolobenzodiazepine derivatives 10a–c:

To an oven-dried screw-capped reaction vial equipped with a magnetic stirring bar 2-azidobenzaldehyde (**1a**, 0.2 mmol), propargylamine (**2a**, 0.2 mmol), 4 Å molecular sieves (50 mg) and ethanol (2 mL) were added. The mixture was stirred at room temperature under argon for one hour. Trichloroacetic acid (**9**, 0.2 mmol) and isocyanide **4a,b,d** (0.2 mmol) were then added and stirred for an additional 23 hours at room temperature under argon atmosphere. The suspension was filtered and the product washed with pentane (100 mL). The Ugi adduct was dried and taken to the next step without further purification. The subsequent ring-closing steps were performed in one-pot starting with the base induced cyclisation. A 1.0 M ethanolic solution of NaOEt was dropped into a well-stirred suspension of Ugi adduct in EtOH (4 mL) from which a precipitation was observed within a few minutes. Upon completion, the reaction mixture was neutralized with 1 N HCl and diluted with 8 mL of EtOH. This mixture was refluxed for 4 hours after which the reaction mixture was concentrated under reduced pressure and the residue on silica gel chromatography (heptane/EtOAc mixtures as eluent) yielded the corresponding hydantoin fused triazolobenzodiazepine.

12-Benzyl-9*H*-benzo[*f*]imidazo[1,5-*d*][1,2,3]triazolo[1,5-*a*][1,4]diazepine-11,13 (12*H*,13a*H*)-dione (**10a**)**:** Yield: 45% (31 mg); White solid - Mp: 126-128 ^{o}C; (^{1}H

NMR, 300 MHz, $CDCl_3$) δ: 4.08 (d, 1H, *J* = 14.4 Hz), 4.70 (s, 2H), 4.90 (s, 1H), 5.10 (d, 1H, *J* = 14.7 Hz), 7.19-7.31 (m, 3H), 7.35-7.39 (m, 2H), 7.46-7.48 (m, 2H), 7.55-7.59 (m, 1H), 7.74 (s, 1H), 7.86 (d, 1H, *J* = 7.8 Hz); (^{13}C NMR, 75 MHz, $CDCl_3$) δ: 34.9, 42.9, 59.3, 124.3, 124.8, 127.1, 128.2, 128.7, 128.8, 130.3, 130.7, 130.9, 133.1, 135.5, 135.5, 135.9, 154.4, 168.3; Exact mass (HRMS, ESI) calculated for $C_{19}H_{15}N_5O_2$ $(M+H)^+$: 346.1304, found: 346.1309.

12-Cyclohexyl-9*H*-benzo[*f*]imidazo[1,5-*d*][1,2,3]triazolo[1,5-*a*][1,4]diazepine-11,13(12*H*,13a *H*)-dione (**10b**): Yield: 82% (82 mg); White solid - Mp: 192-194 °C; (^{1}H NMR, 300 MHz, $CDCl_3$) δ: 1.23-1.43 (m, 4H), 1.68-190 (m, 4H), 2.12-2.29 (m, 2H), 4.0-4.07 (m, 1H), 4.14 (d, 1H, *J* = 14.4 Hz), 4.85 (s, 1H), 5.16 (d, 1H, *J* = 14.7 Hz), 7.58-7.59 (m, 2H), 7.63-7.69 (m, 1H), 7.84 (s, 1H), 7.96 (d, 1H, *J* = 7.8 Hz); (^{13}C NMR, 75 MHz, $CDCl_3$) δ: 24.9, 25.8, 29.3, 29.6, 30.3, 34.9, 52.3, 58.6, 124.7, 124.8, 127.1, 130.2, 130.7, 130.8, 133.1, 135.9, 154.5, 168.5; Exact mass (HRMS, ESI) calculated for $C_{18}H_{19}N_5O_2$ $(M+H)^+$: 338.1617, found: 338.1621.

12-(4-Methoxyphenyl)-9*H*-benzo[*f*]imidazo[1,5-*d*][1,2,3]triazolo[1,5-*a*][1,4]diazepine-11,13(12*H*,13a*H*)-dione (**10c**): Yield: 62% (45 mg); White solid - Mp: 229-232 °C; (^{1}H NMR, 300 MHz, $CDCl_3$) δ: 3.85 (s, 3H), 4.24 (d, *J*=14.7, 1H), 5.11 (s, 1H), 5.27 (d, *J*=14.7, 1H), 7.05 – 6.98 (m, 2H), 7.39 – 7.32 (m, 2H), 7.64 – 7.57 (m, 1H), 7.73 – 7.66 (m, 2H), 7.87 (d, *J*=0.5, 1H), 7.98 (dd, *J*=8.2, 1.2, 1H); ^{13}C NMR (75 MHz, $CDCl_3$) δ: 35.30, 55.70, 59.22, 114.72, 123.77, 124.51, 124.97, 127.36, 127.64, 130.46, 130.72, 131.19, 133.36, 136.05, 153.75, 159.75, 167.84; Exact mass (HRMS, ESI) calculated for $C_{19}H_{15}N_5O_3$ $(M+H)^+$: 362.1248, found: 362.1245.

5a (^{1}H NMR, 300 MHz, $CDCl_3$)

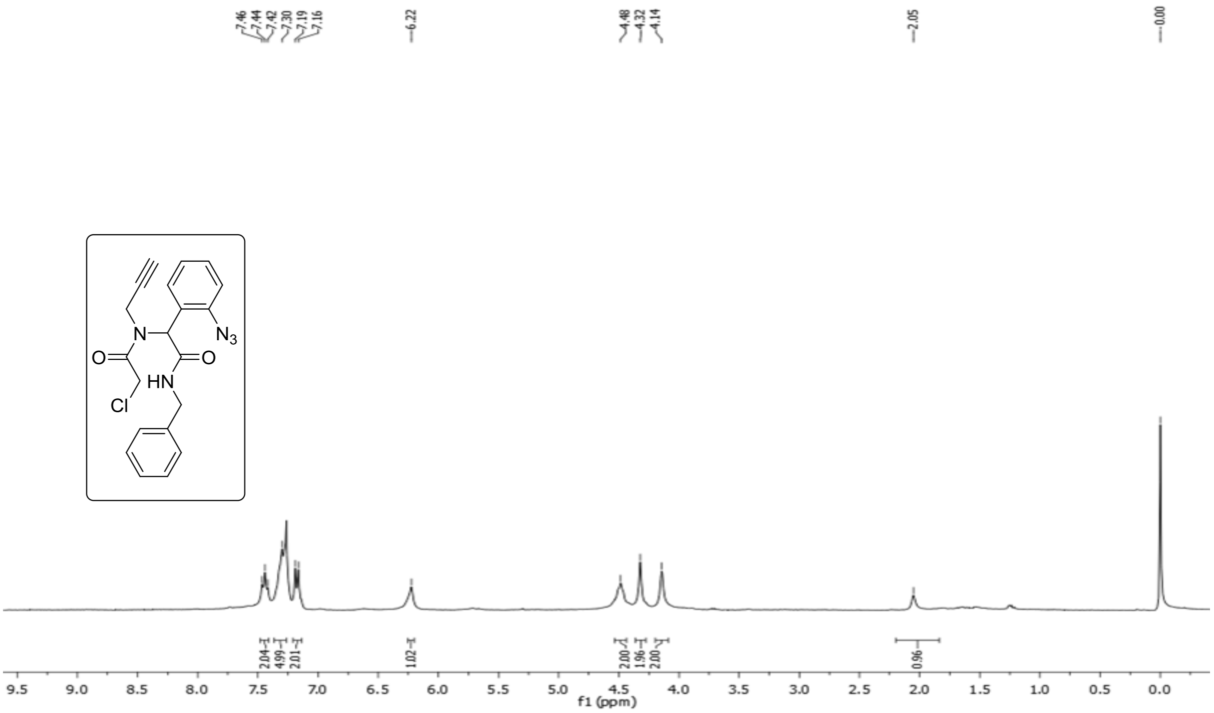

5a (^{1}H NMR, 600 MHz, CD_3CN)

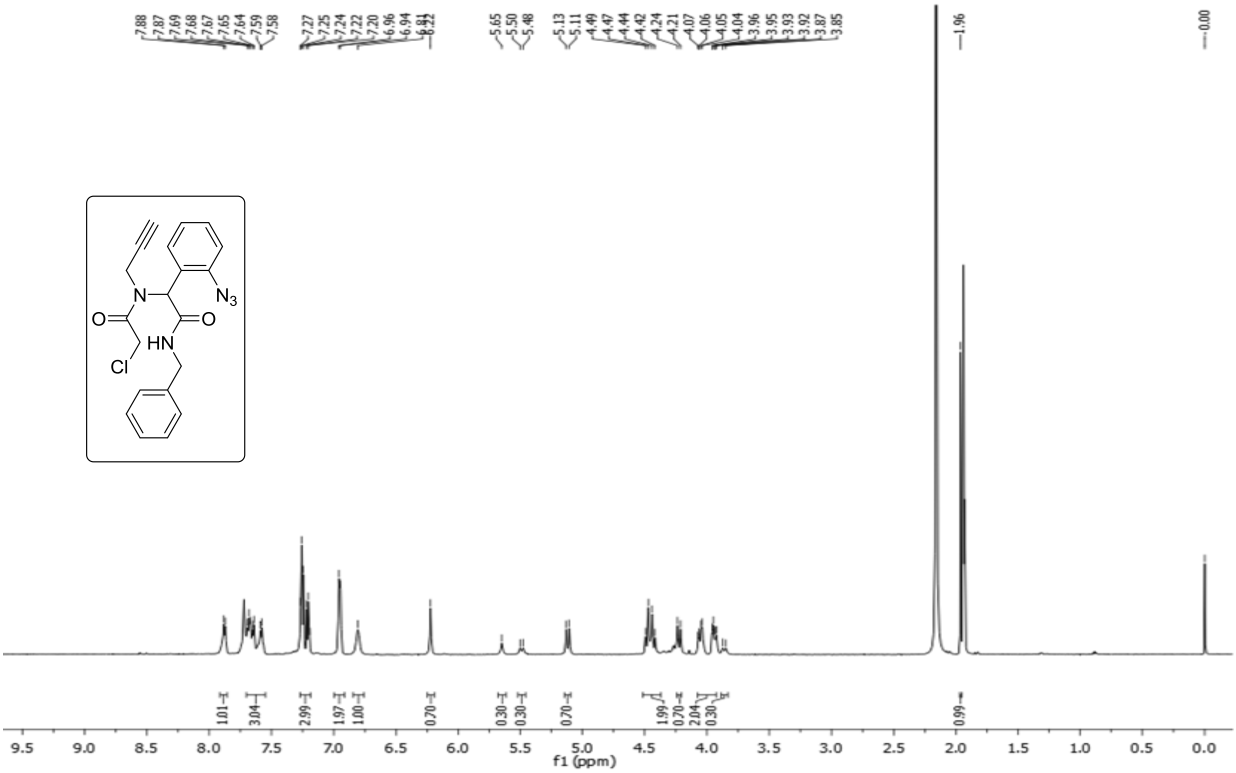

5a (^{13}C NMR, 150 MHz, CD_3CN)

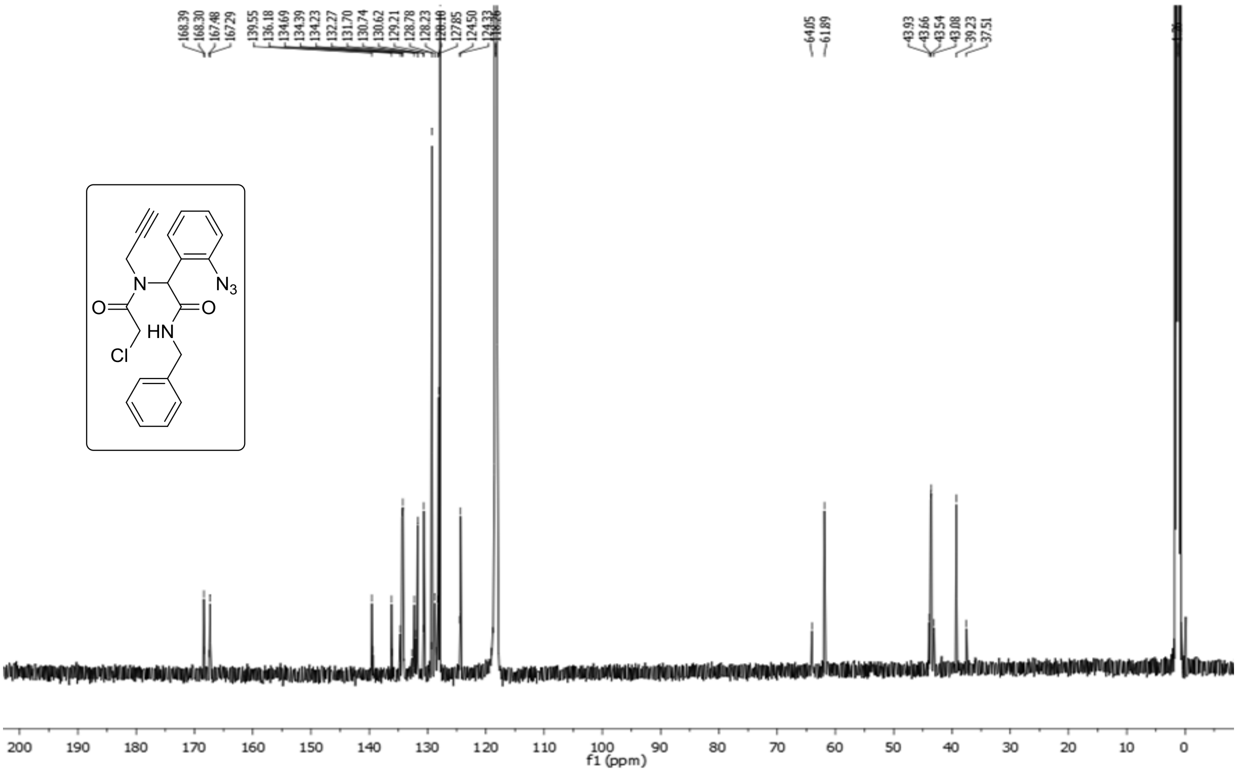

6 (^{1}H NMR, 300 MHz)

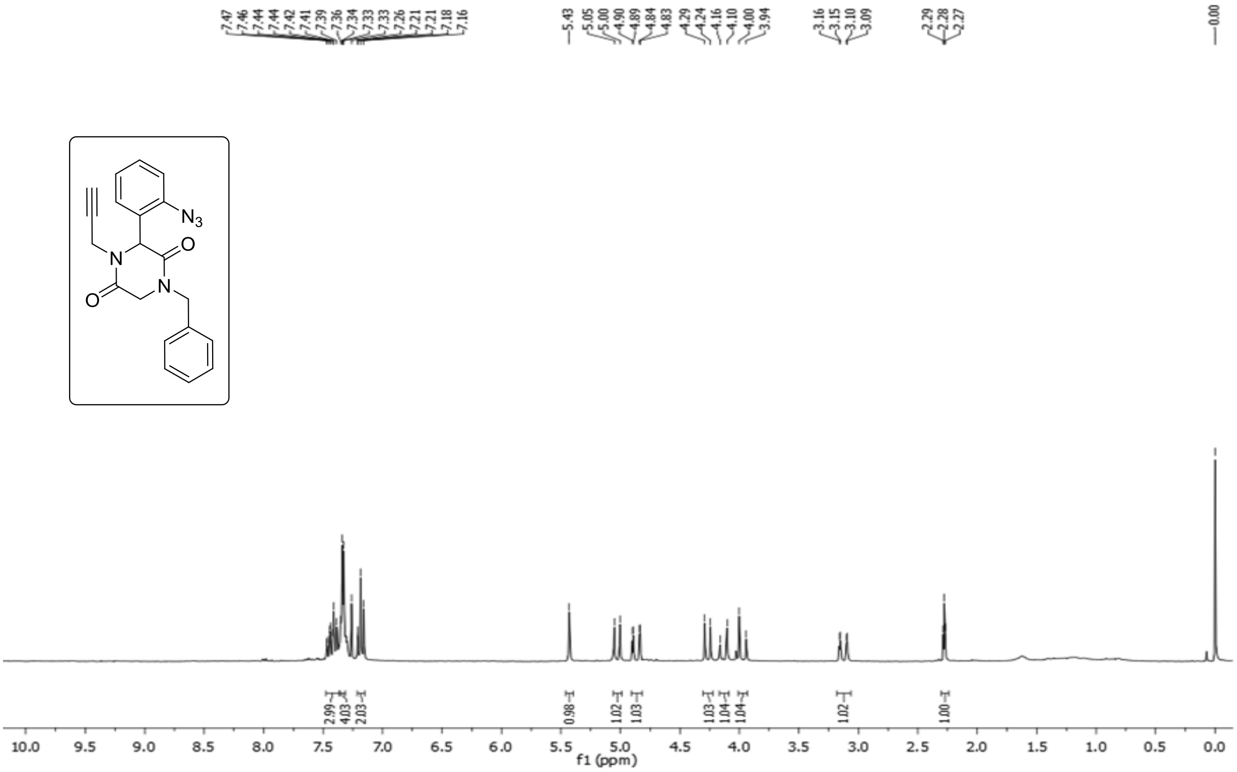

6 (^{13}C NMR, 75 MHz)

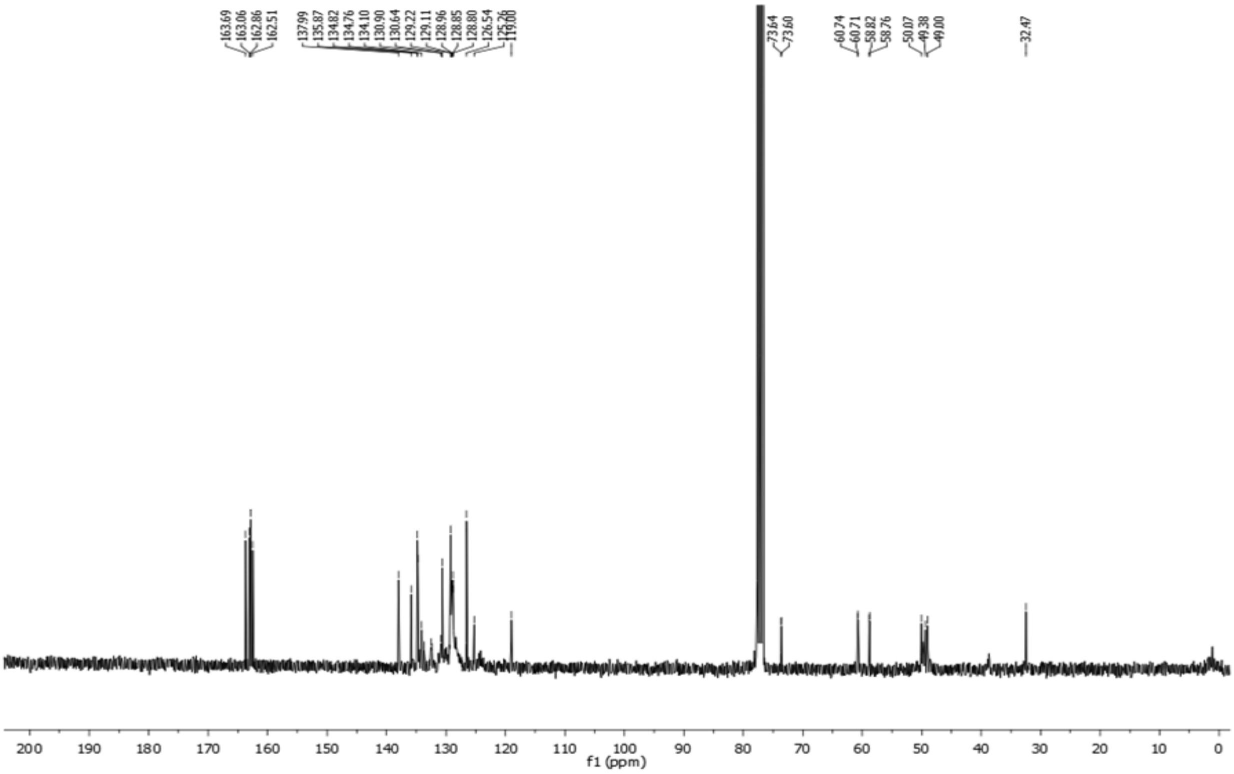

7a (^{1}H NMR, 600 MHz)

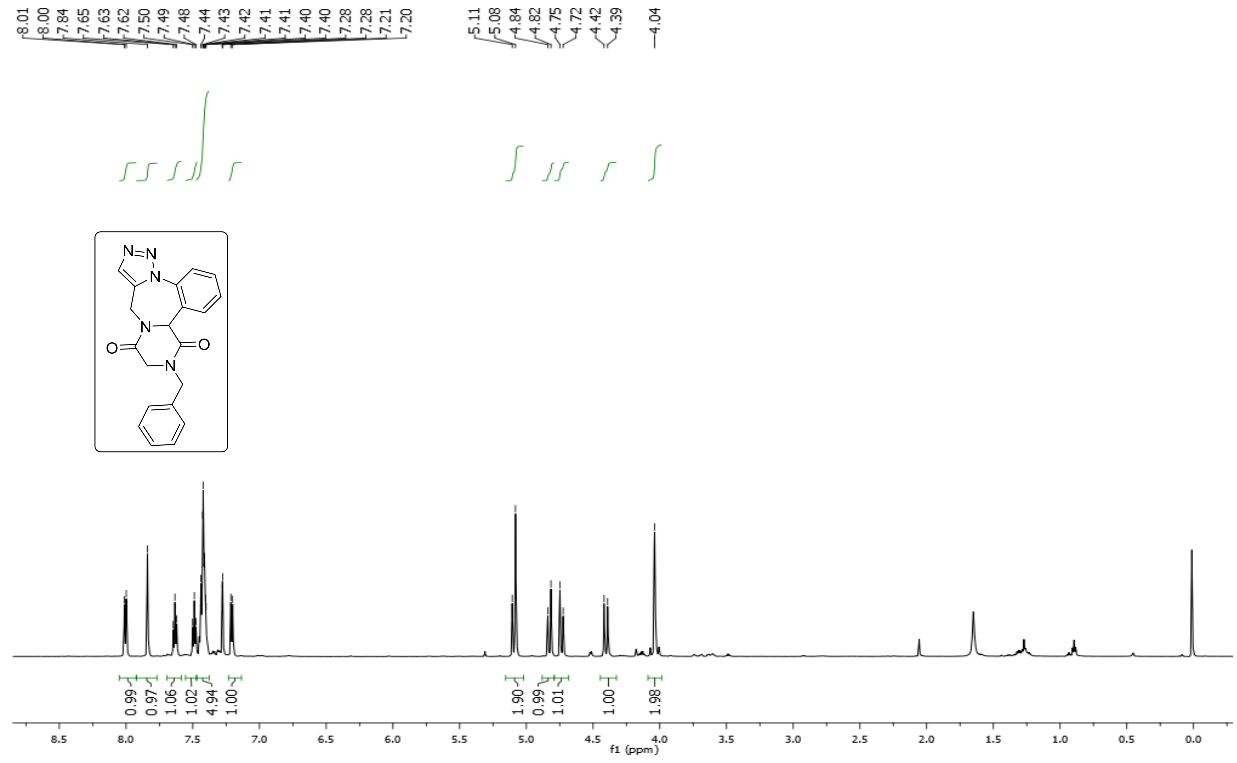

7a (^{13}C NMR, 150 MHz)

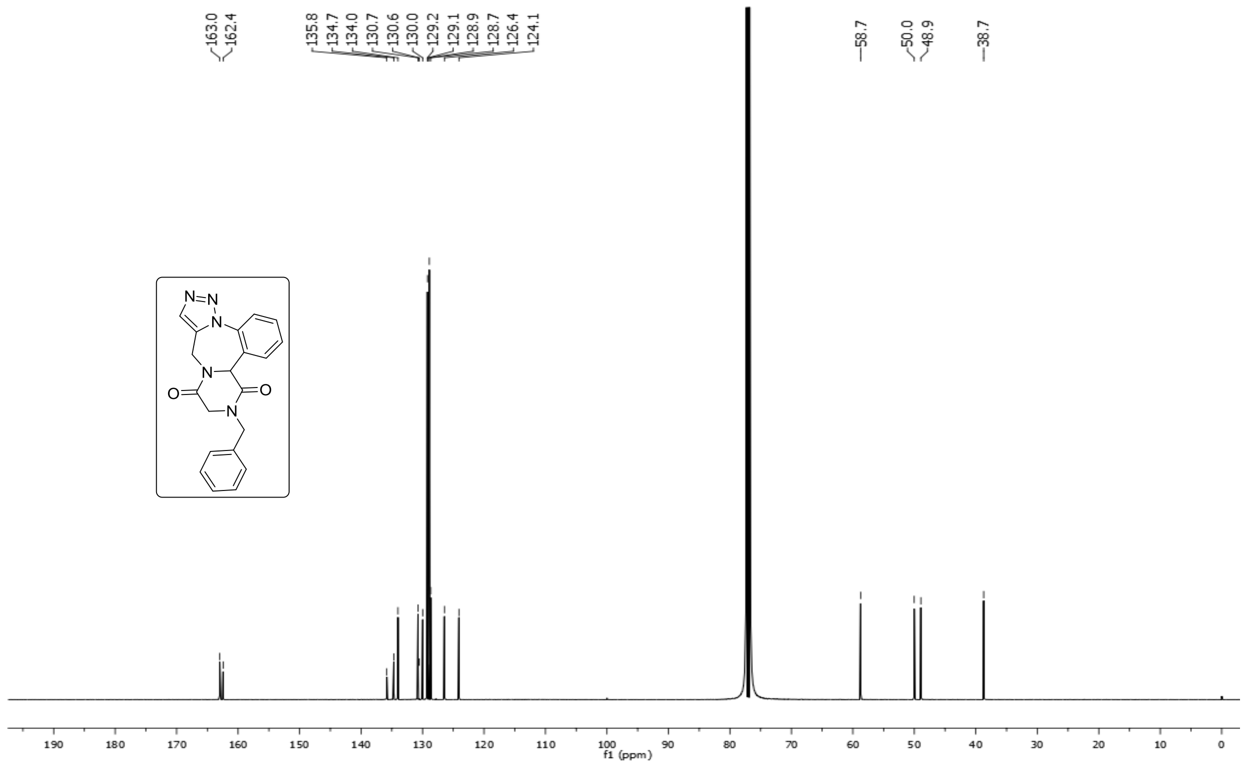

8 (^{1}H NMR, 600 MHz)

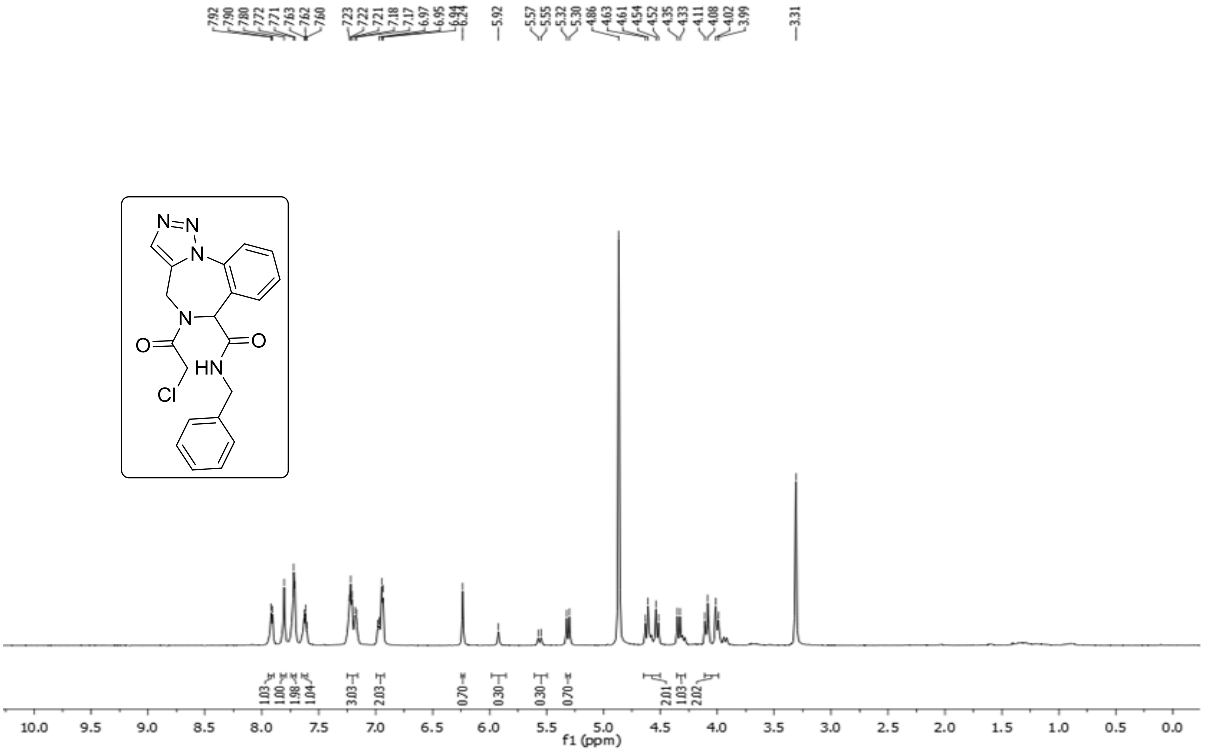

8 (^{13}C NMR, 75 MHz)

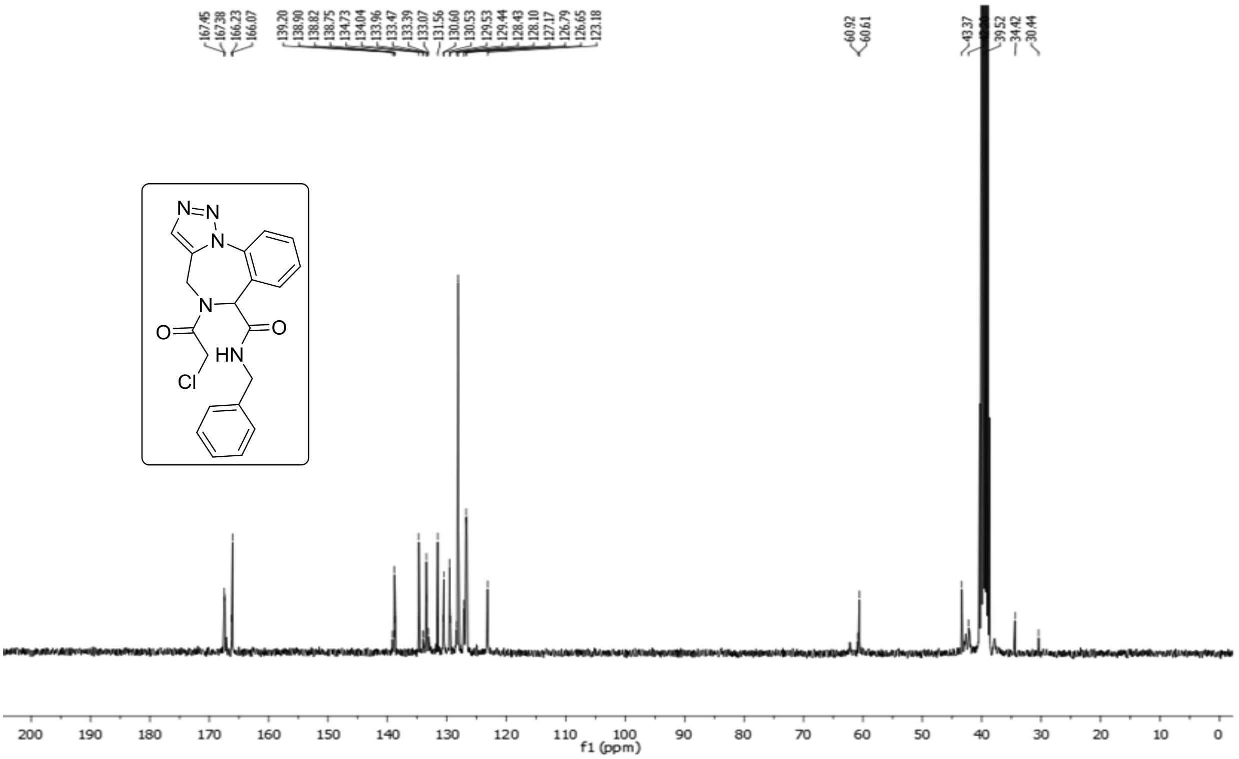

7b (^{1}H NMR, 600 MHz)

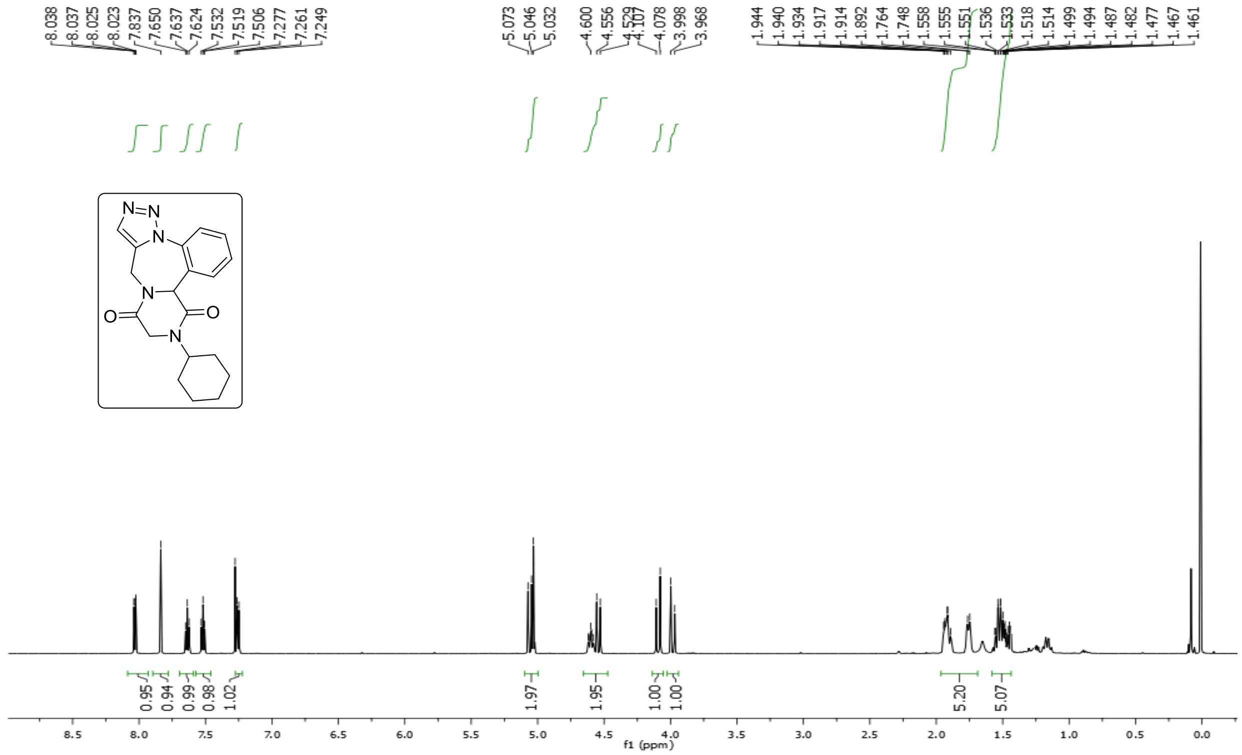

7b (^{13}C NMR, 150 MHz)

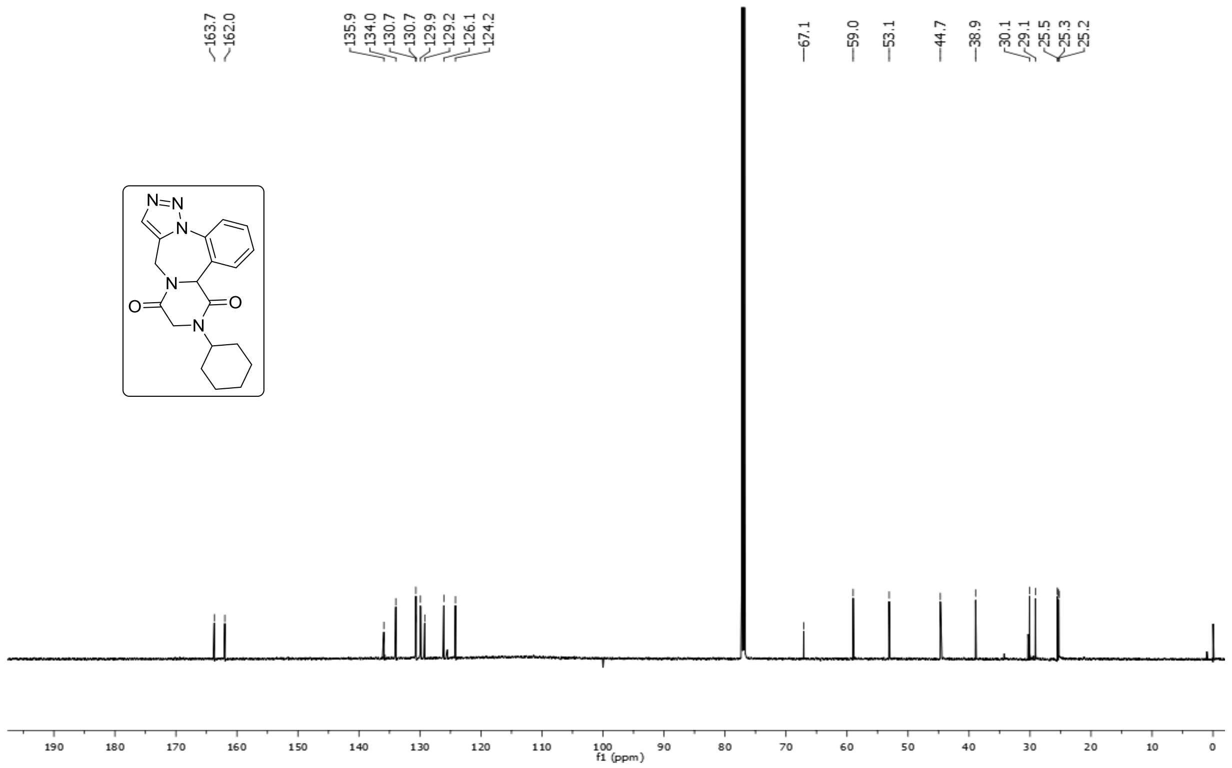

7c (^{1}H NMR, 300 MHz)

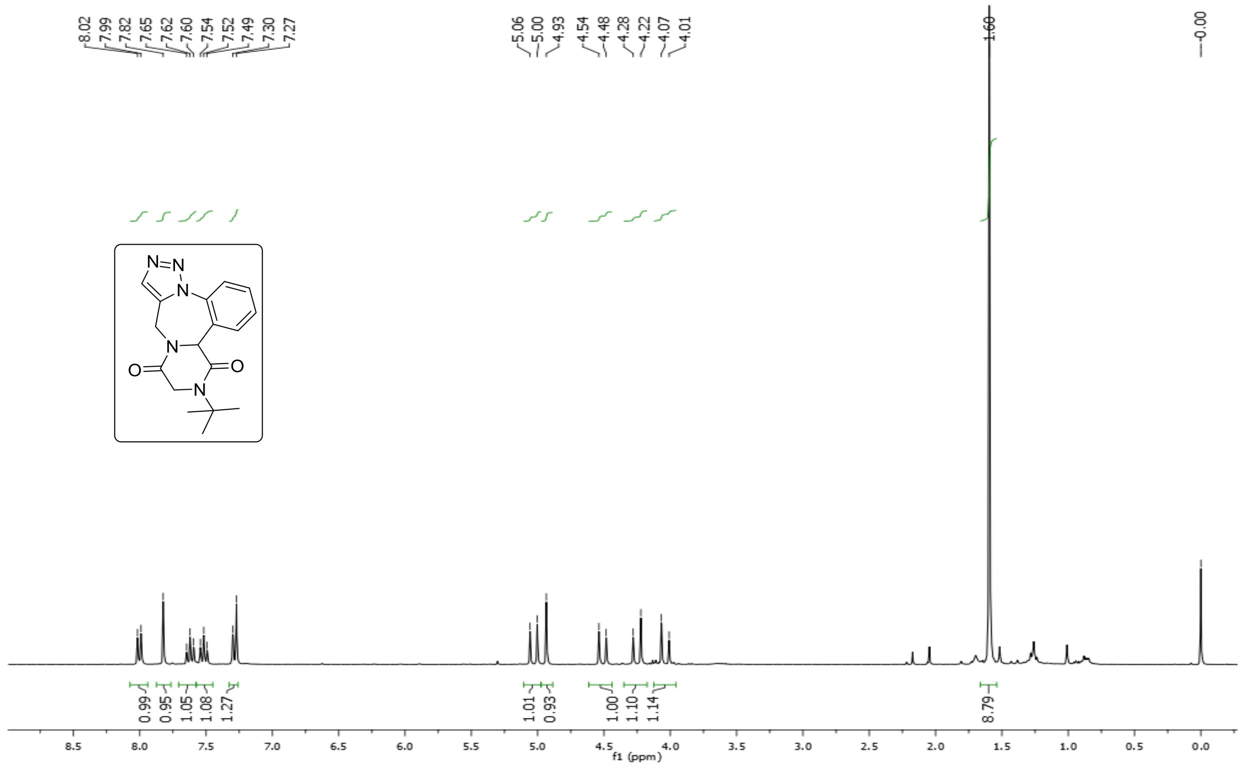

7c (^{13}C NMR, 75 MHz)

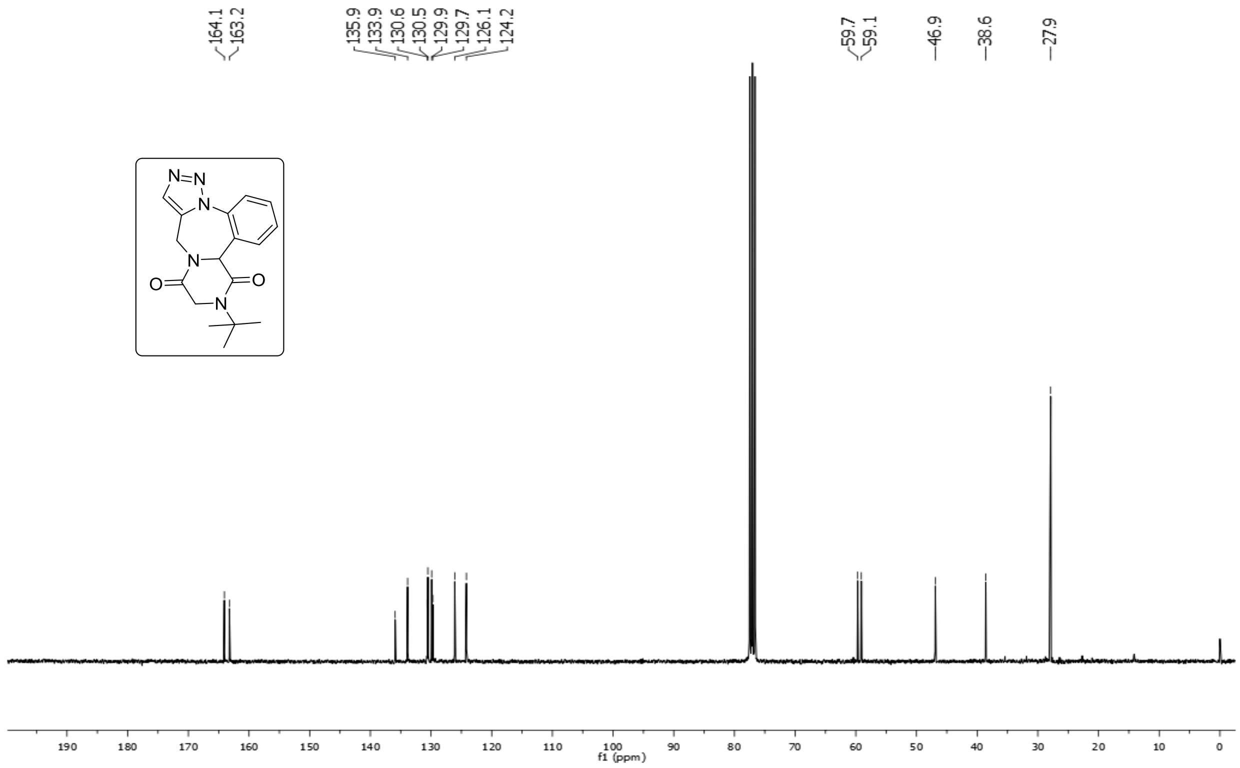

7d (^{1}H NMR, 300 MHz)

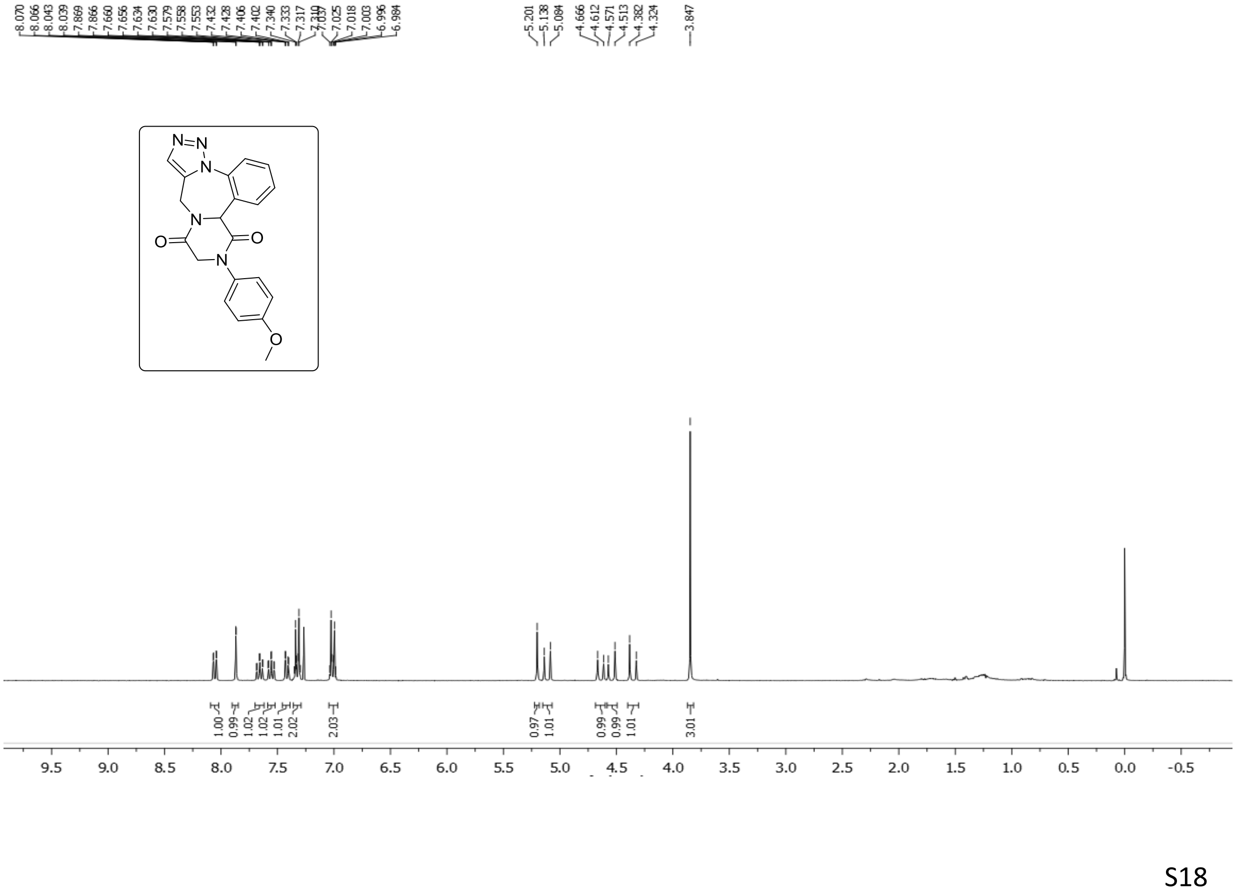

7d (^{13}C NMR, 75 MHz)

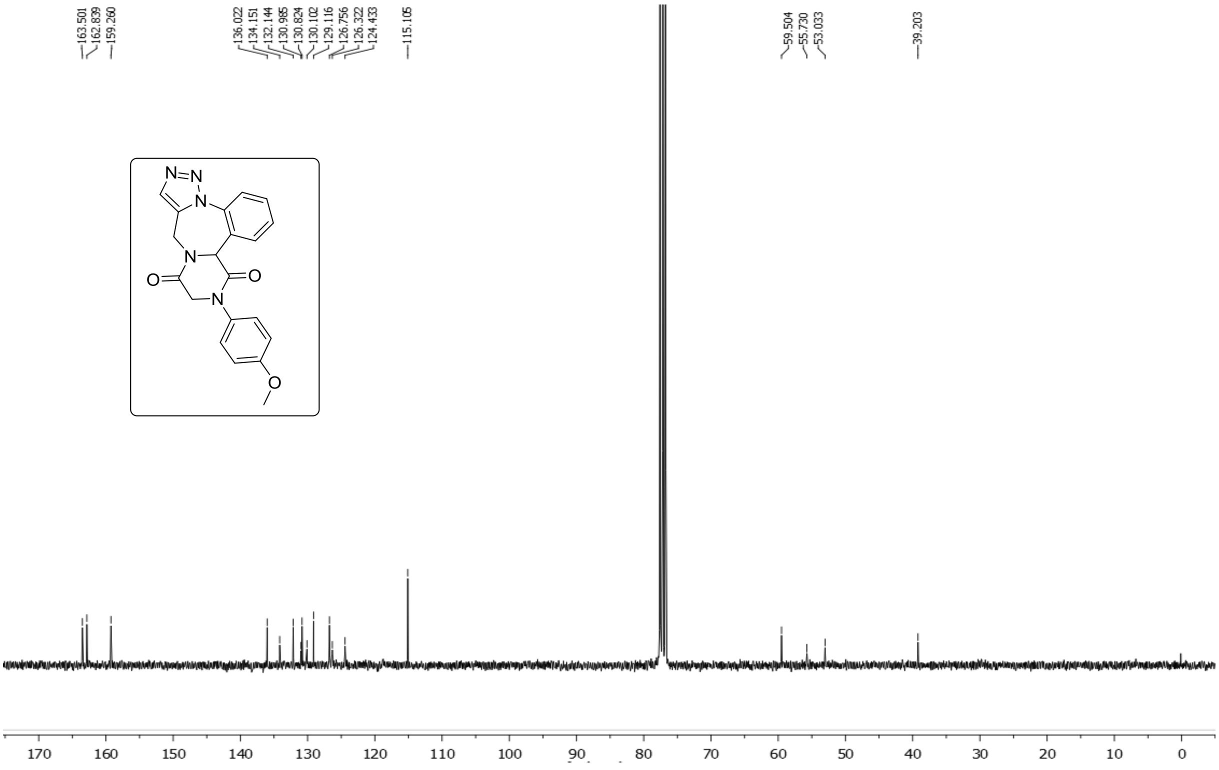

7e (^{1}H NMR, 300 MHz)

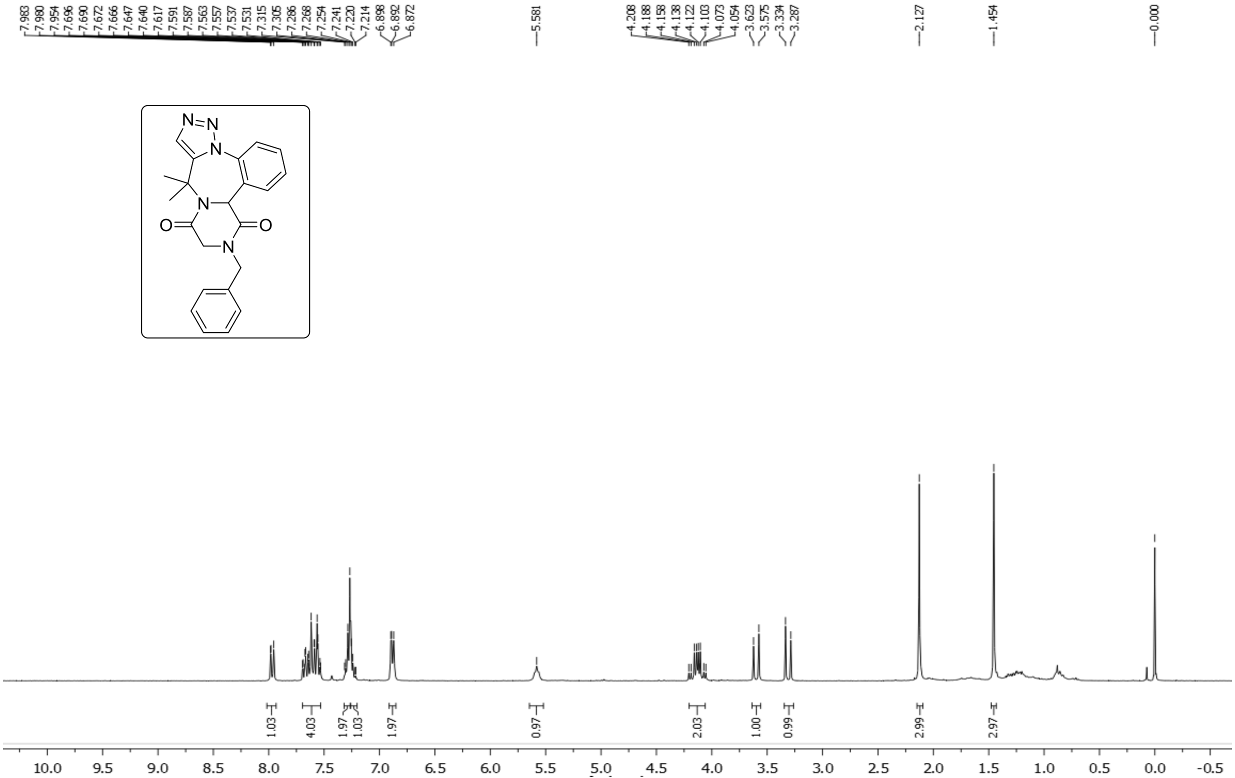

7e (^{13}C NMR, 75 MHz)

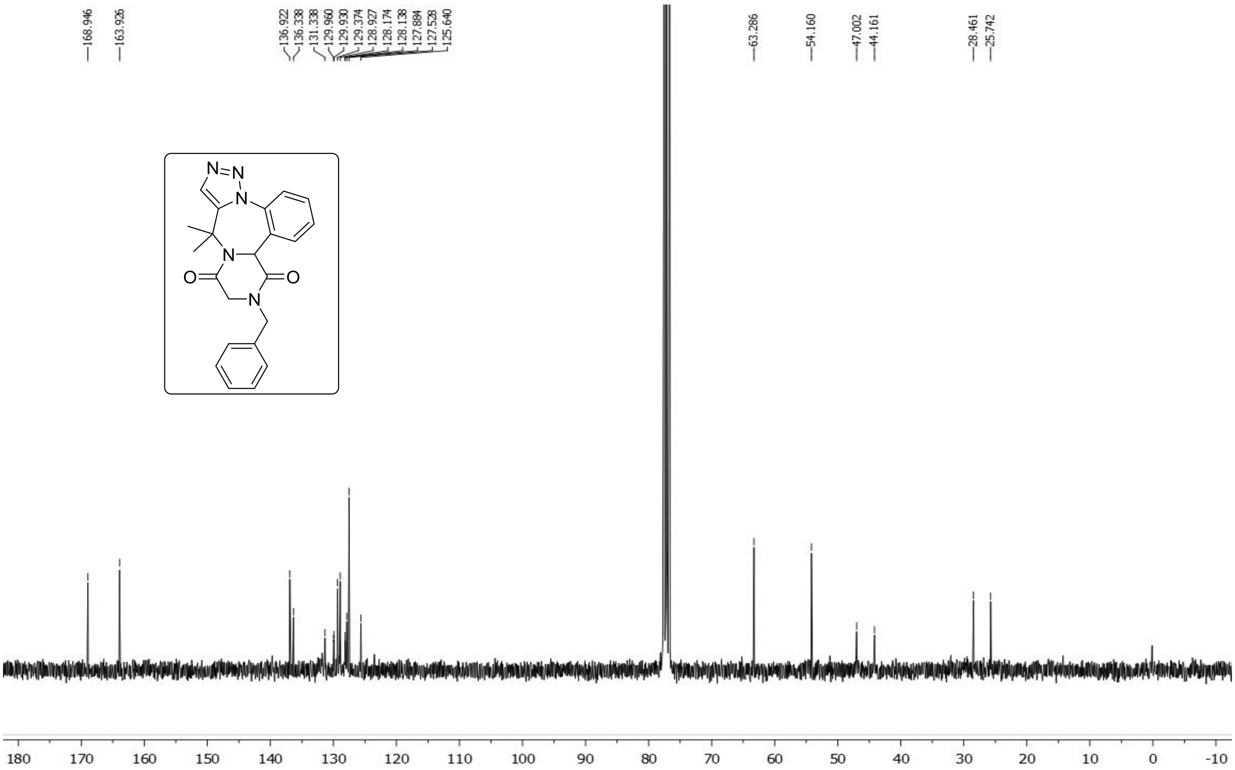

7f (^{1}H NMR, 600 MHz)

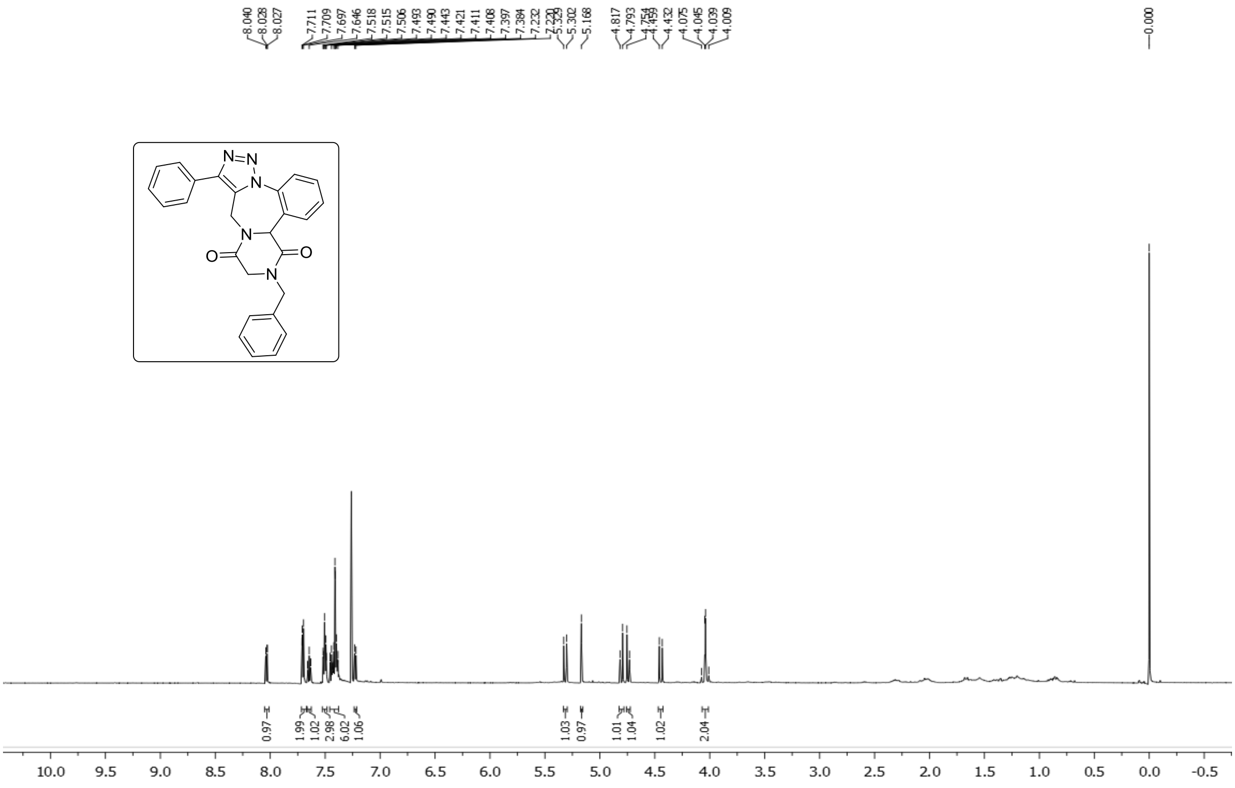

7f (^{13}C NMR, 151 MHz)

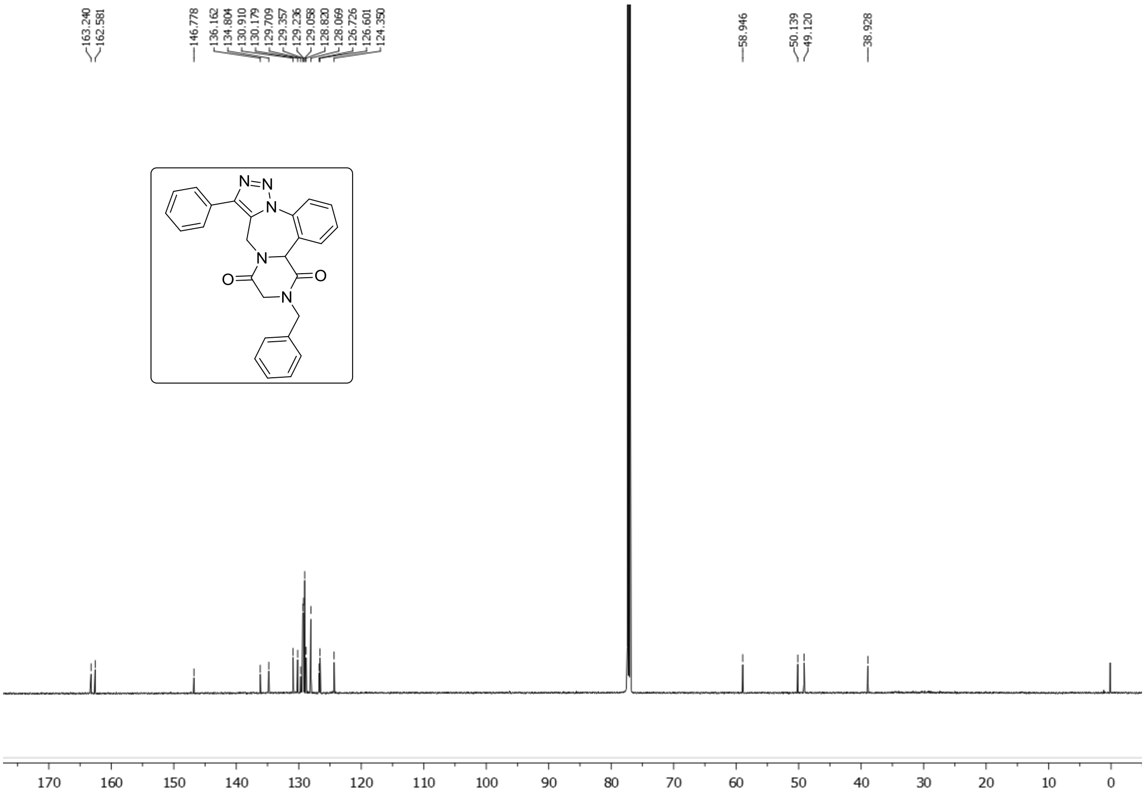

7g (^{1}H NMR, 300 MHz)

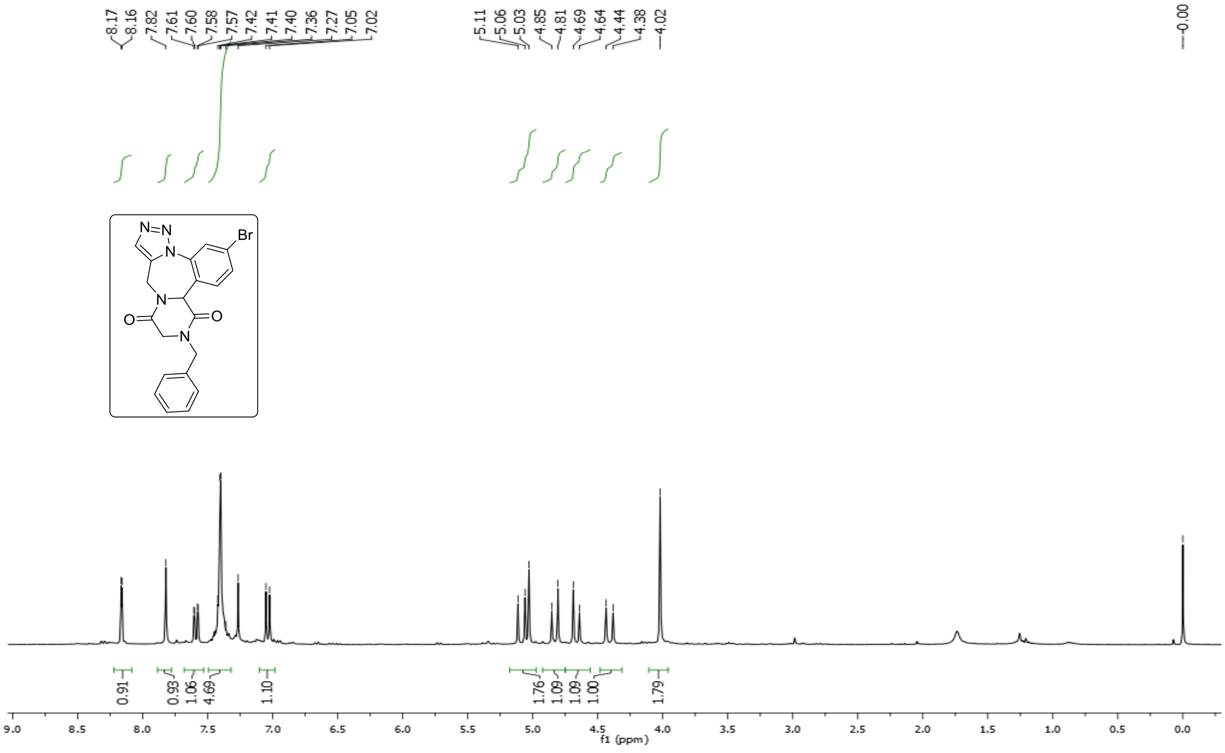

7g (^{13}C NMR, 150 MHz)

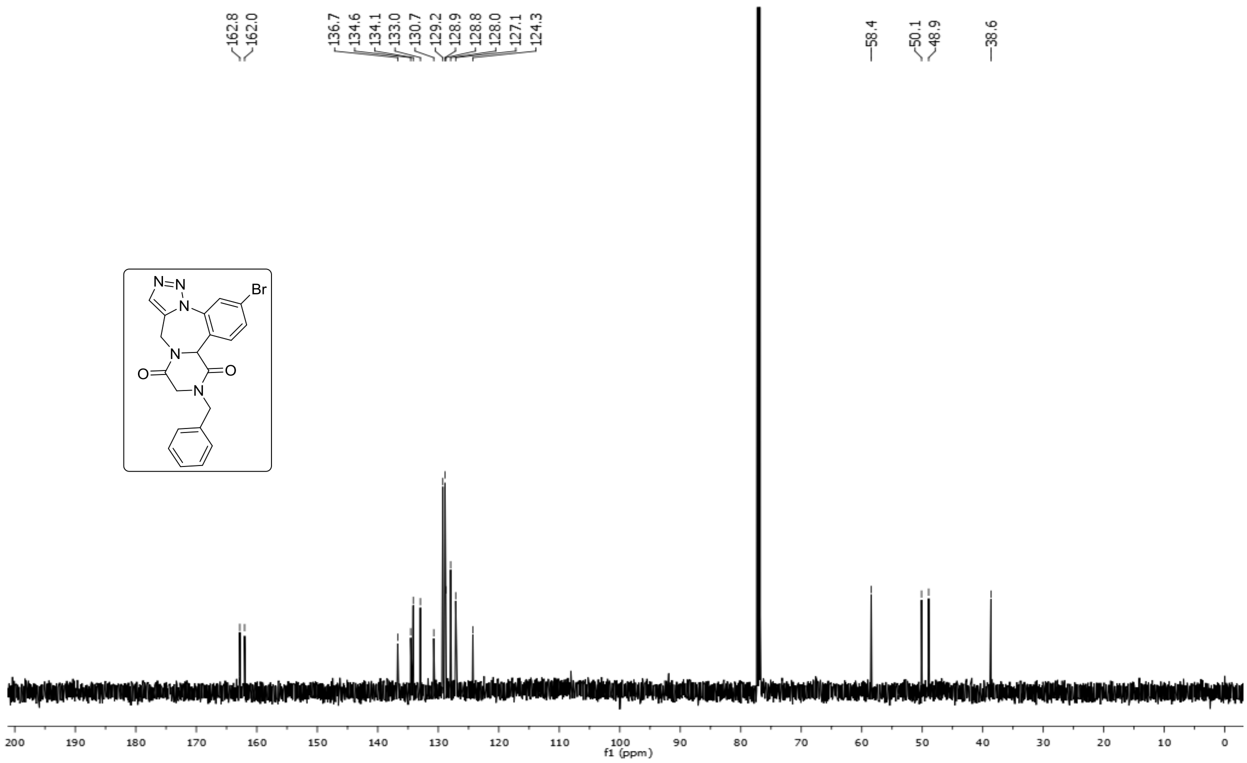

7h (^{1}H NMR, 300 MHz)

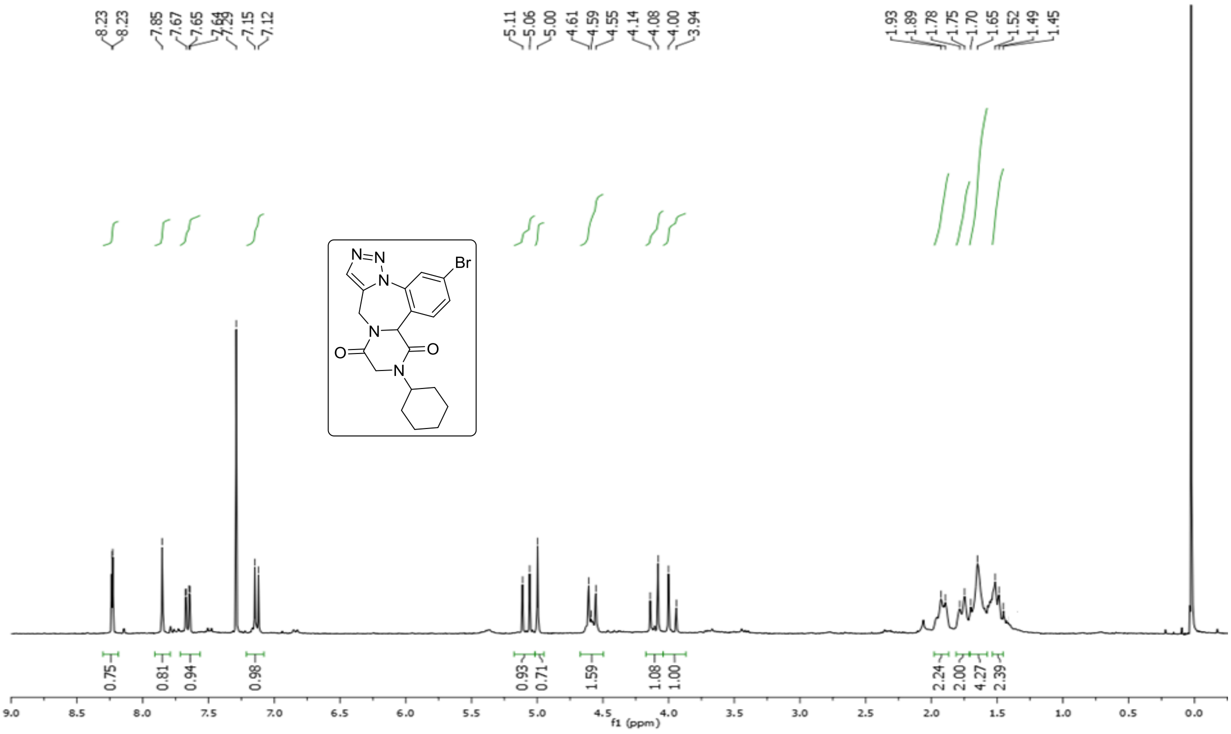

7h (^{13}C NMR, 75 MHz)

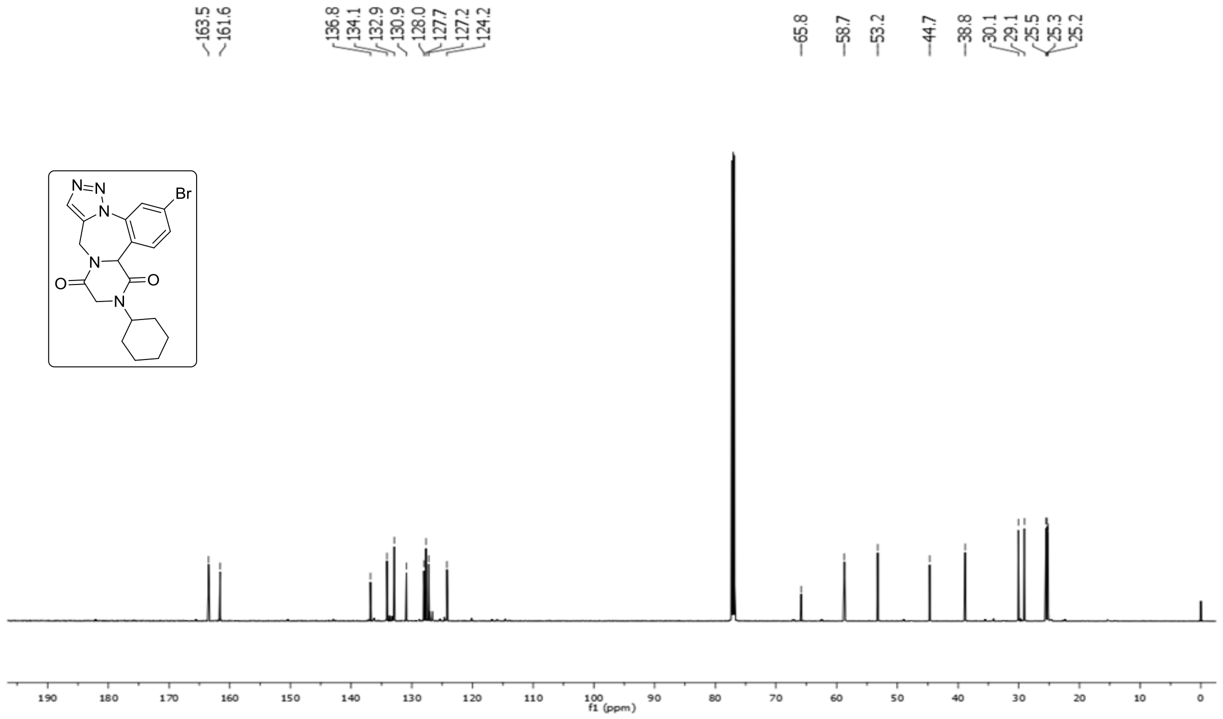

7i (^{1}H NMR, 300 MHz)

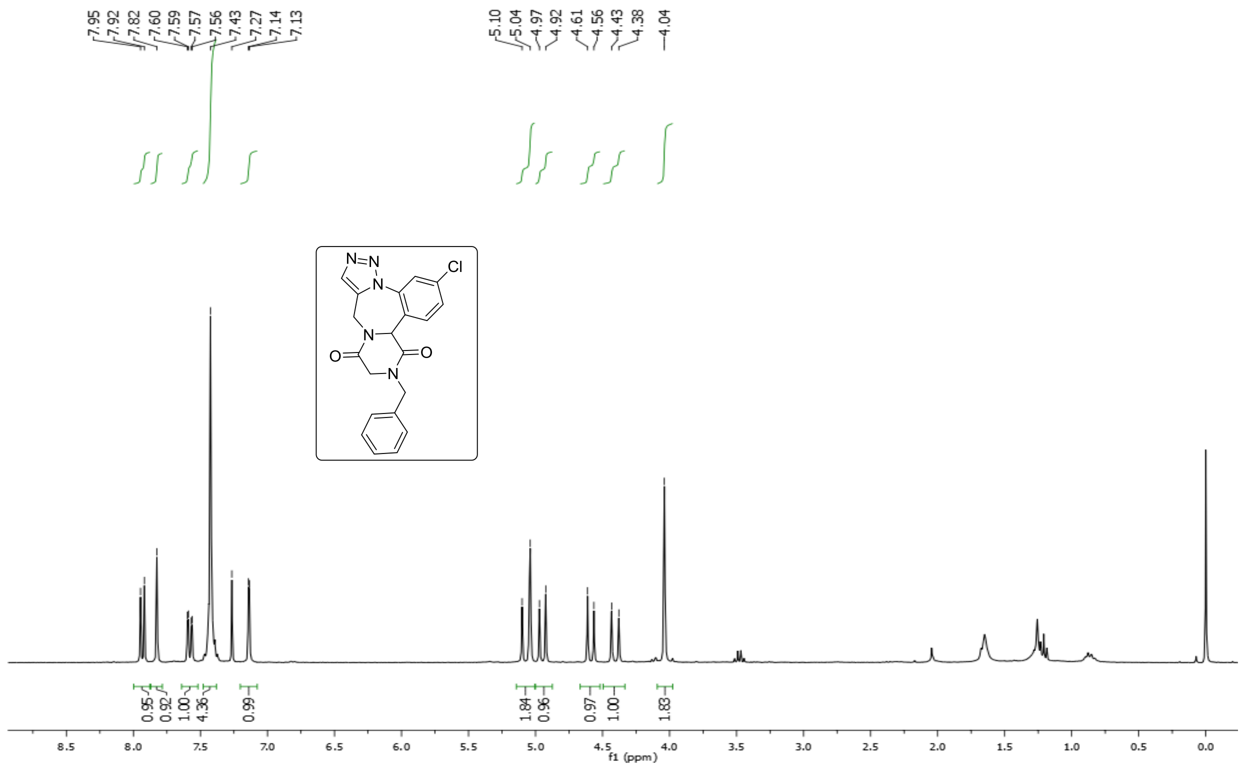

7i (^{13}C NMR, 75 MHz)

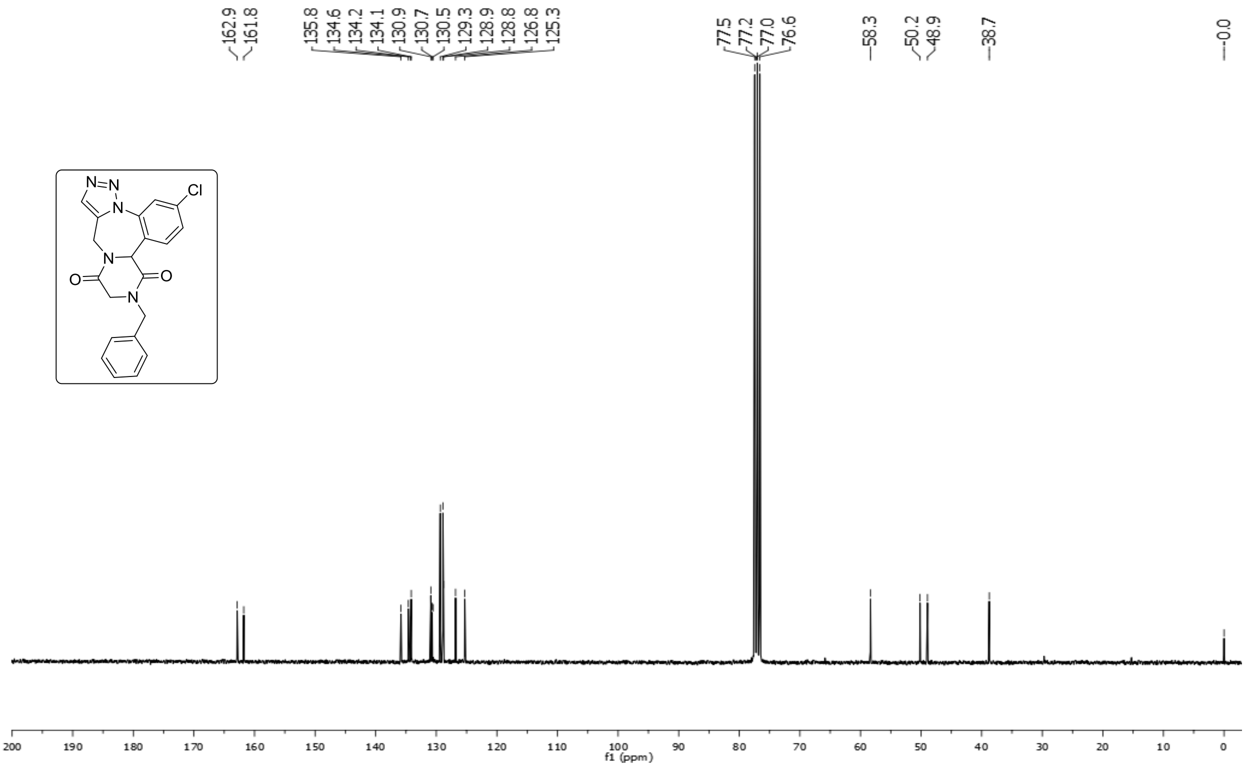

7j (^{1}H NMR, 300 MHz)

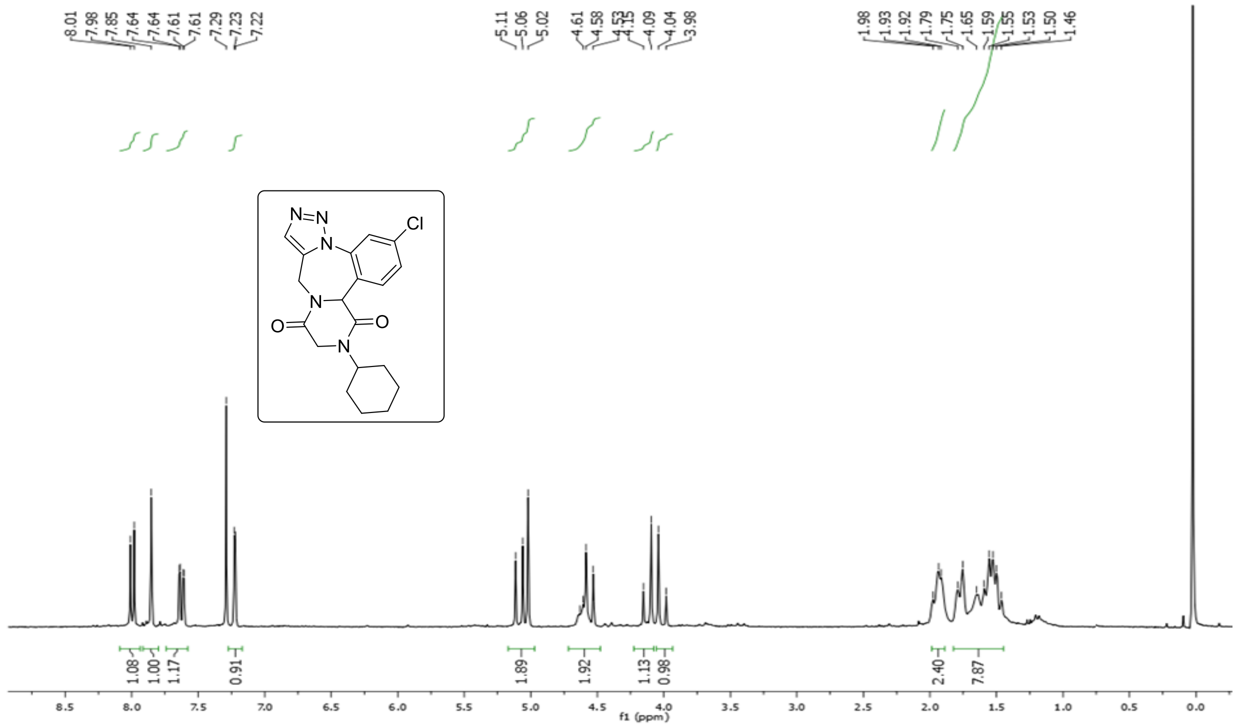

7j (^{13}C NMR, 150 MHz)

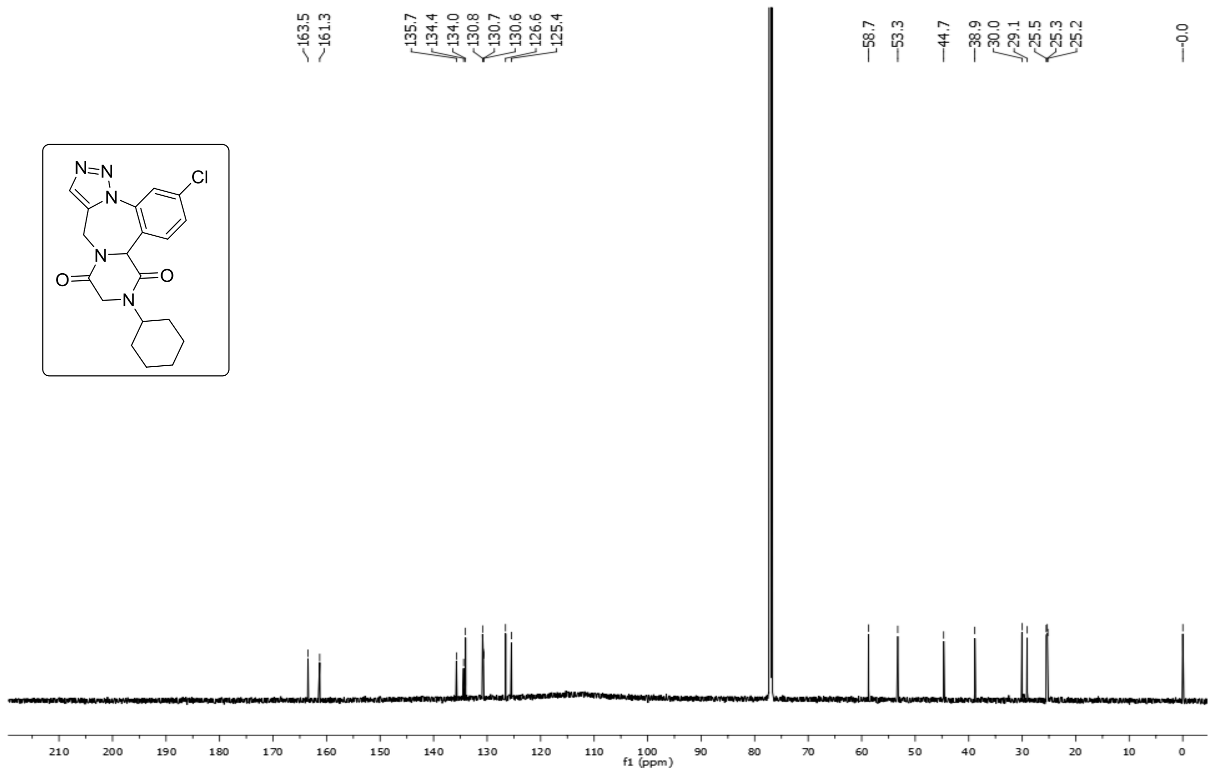

7k (^{1}H NMR, 300 MHz)

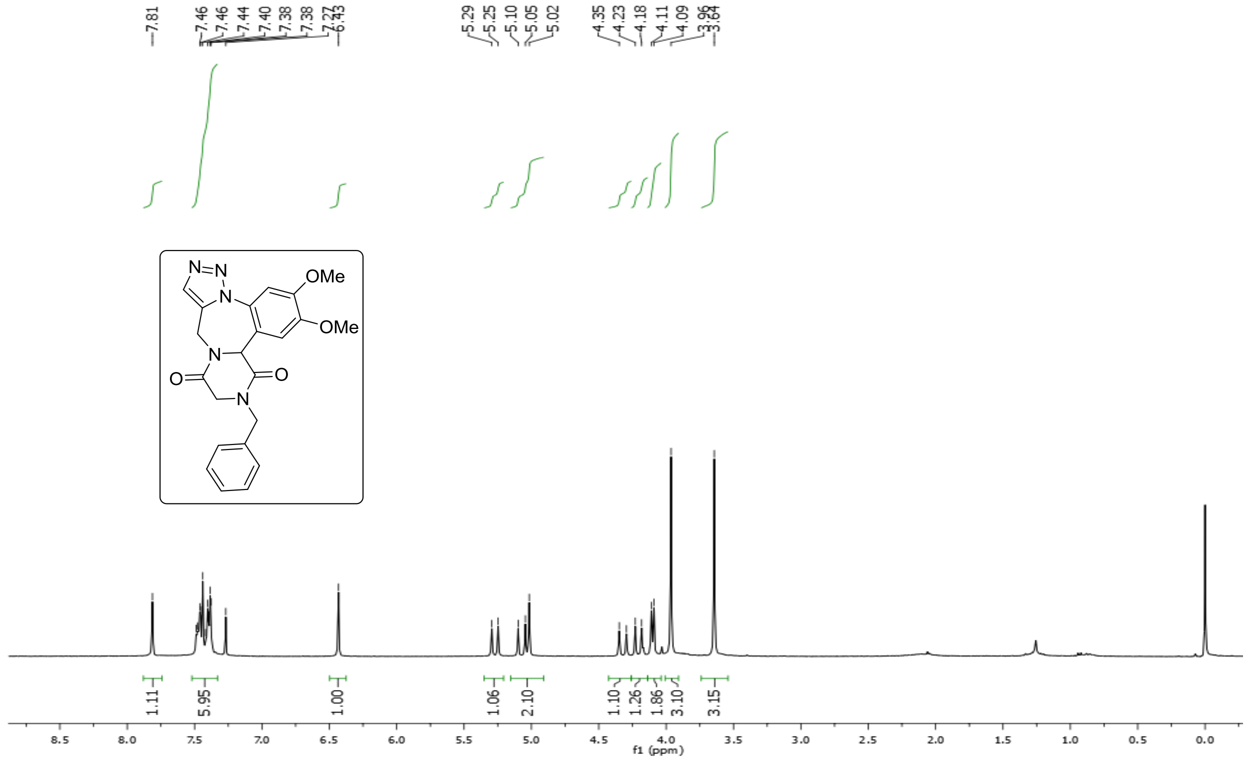

7k (^{13}C NMR, 75 MHz)

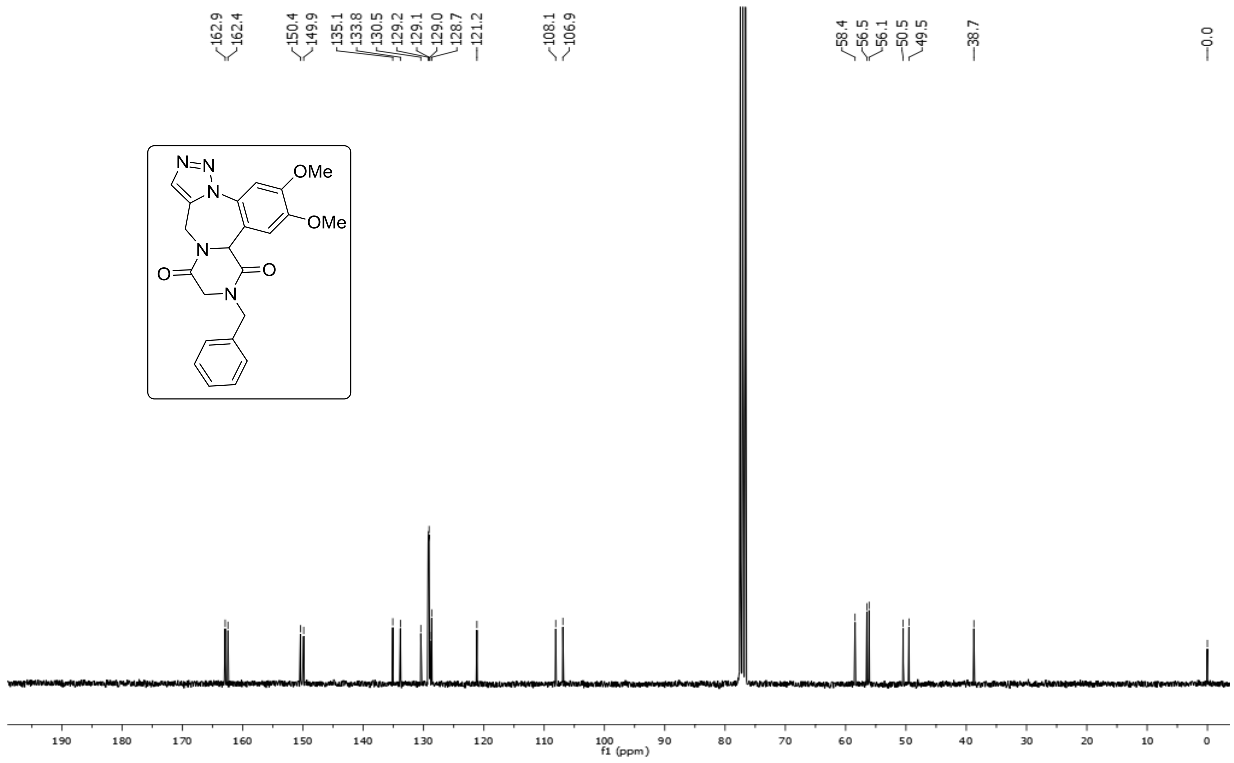

7l (^{1}H NMR, 300 MHz)

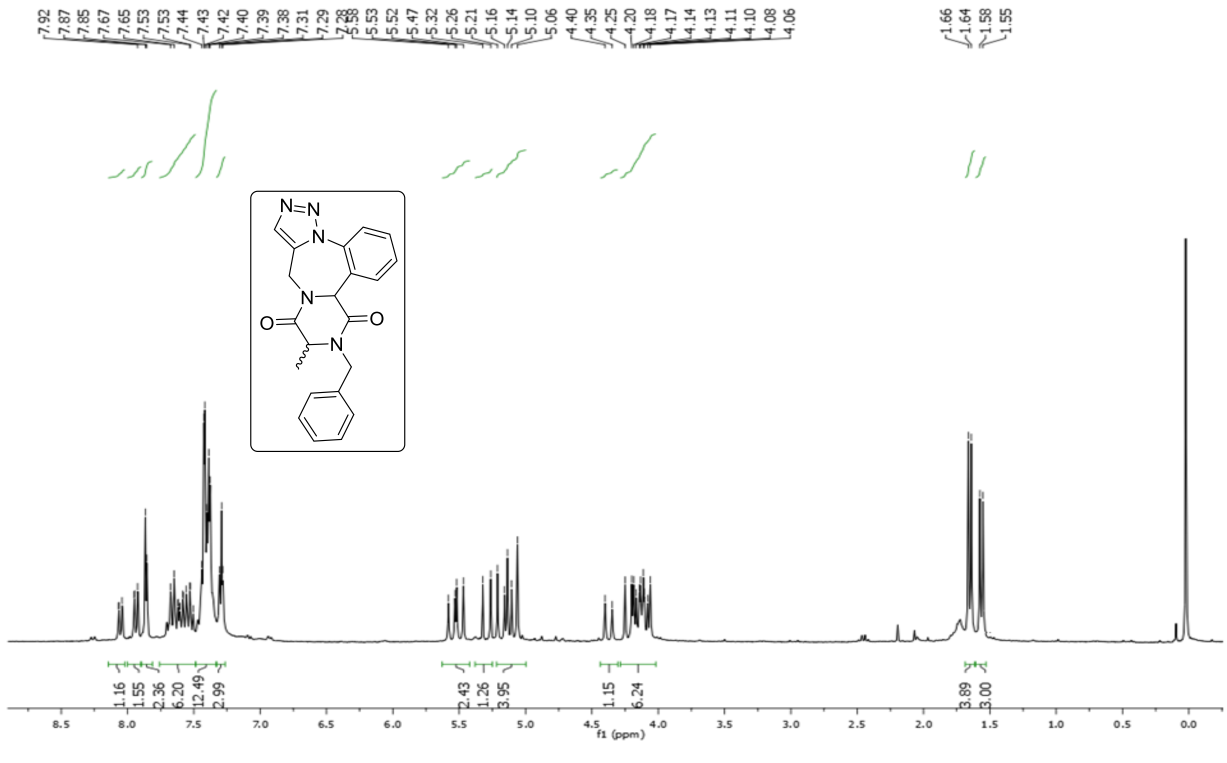

7l (^{13}C NMR, 150 MHz)

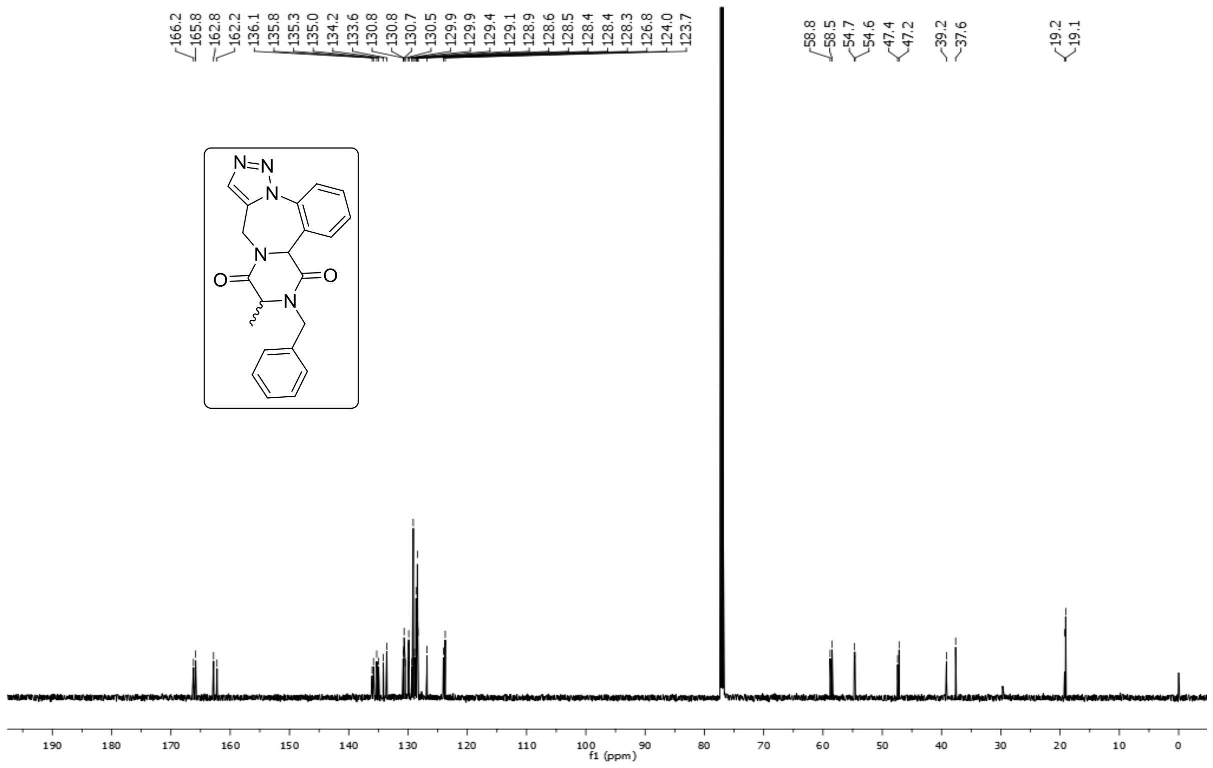

10a (^{1}H NMR, 300 MHz)

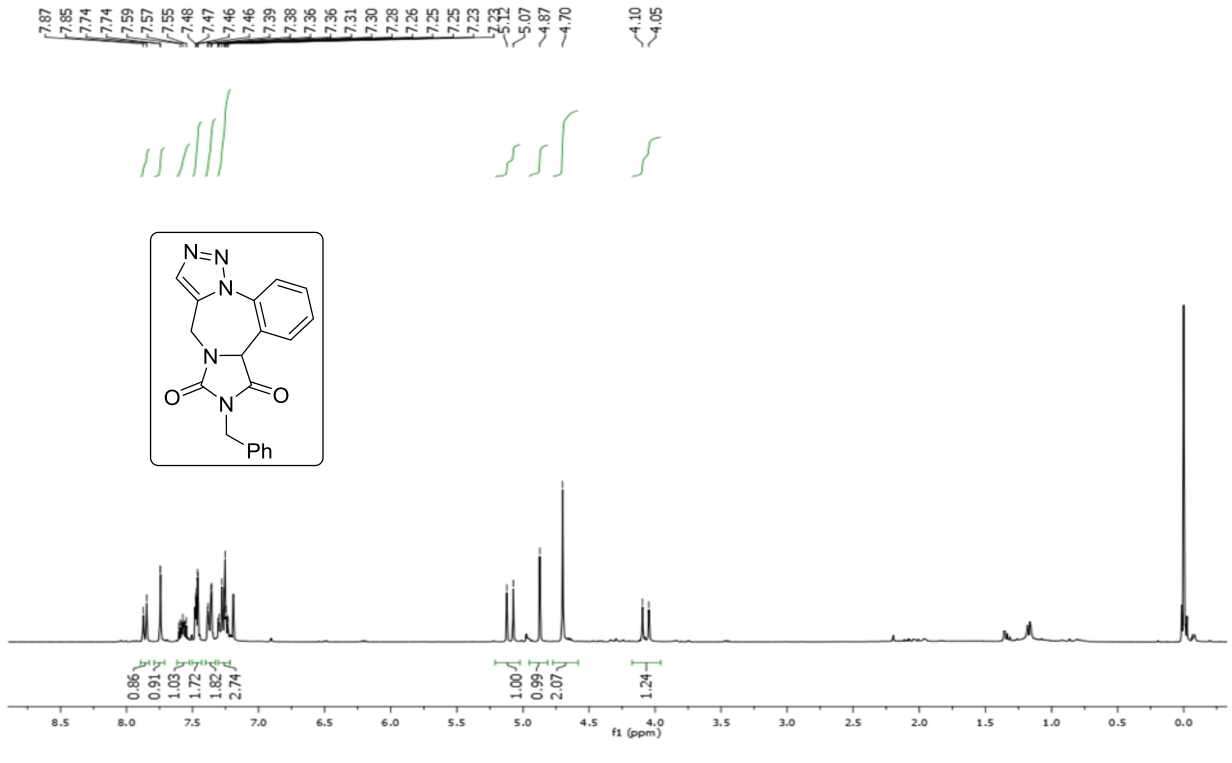

10a (^{13}C NMR, 75 MHz)

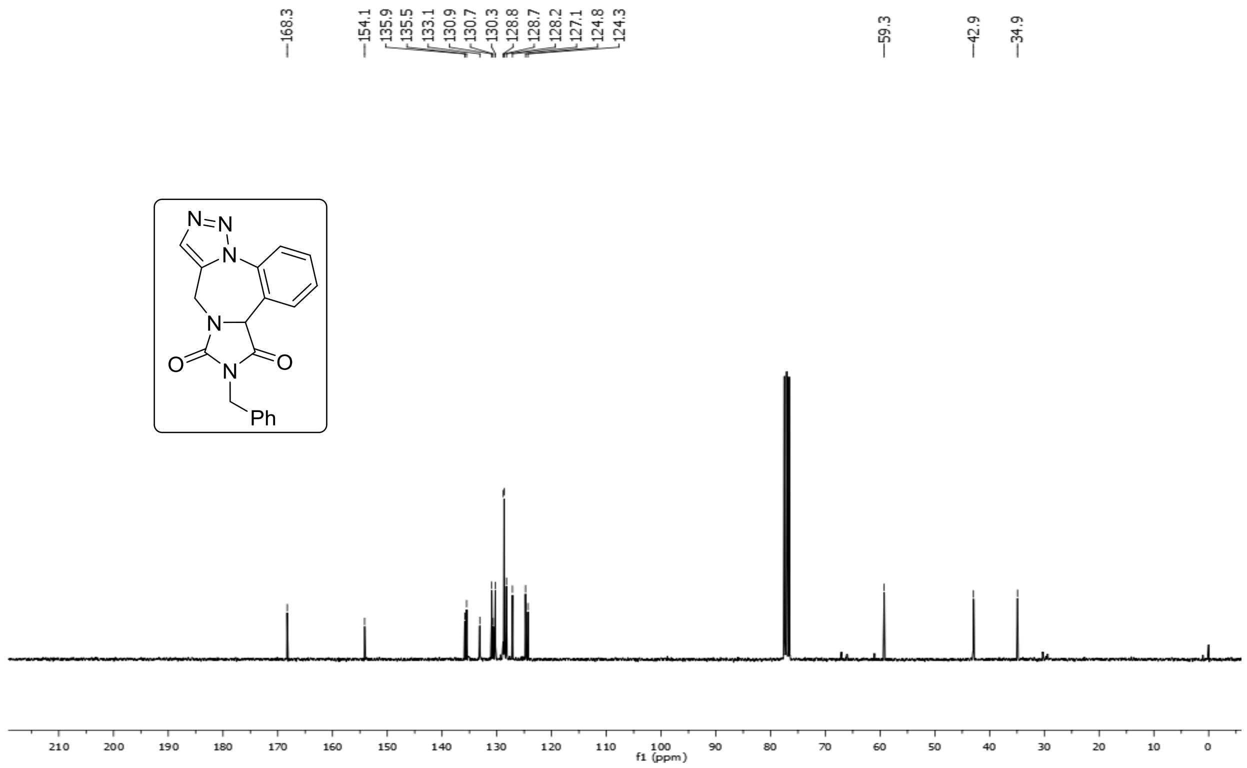

10b (^{1}H NMR, 300 MHz)

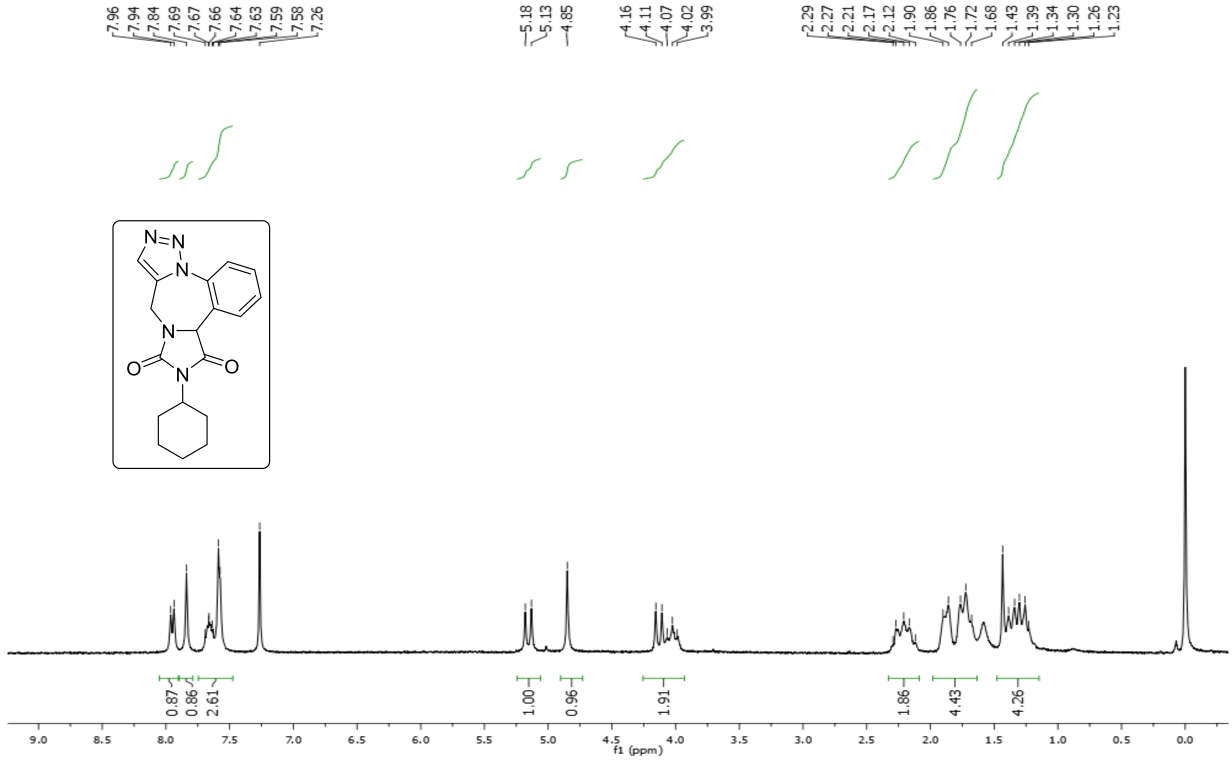

10b (^{13}C NMR, 75 MHz)

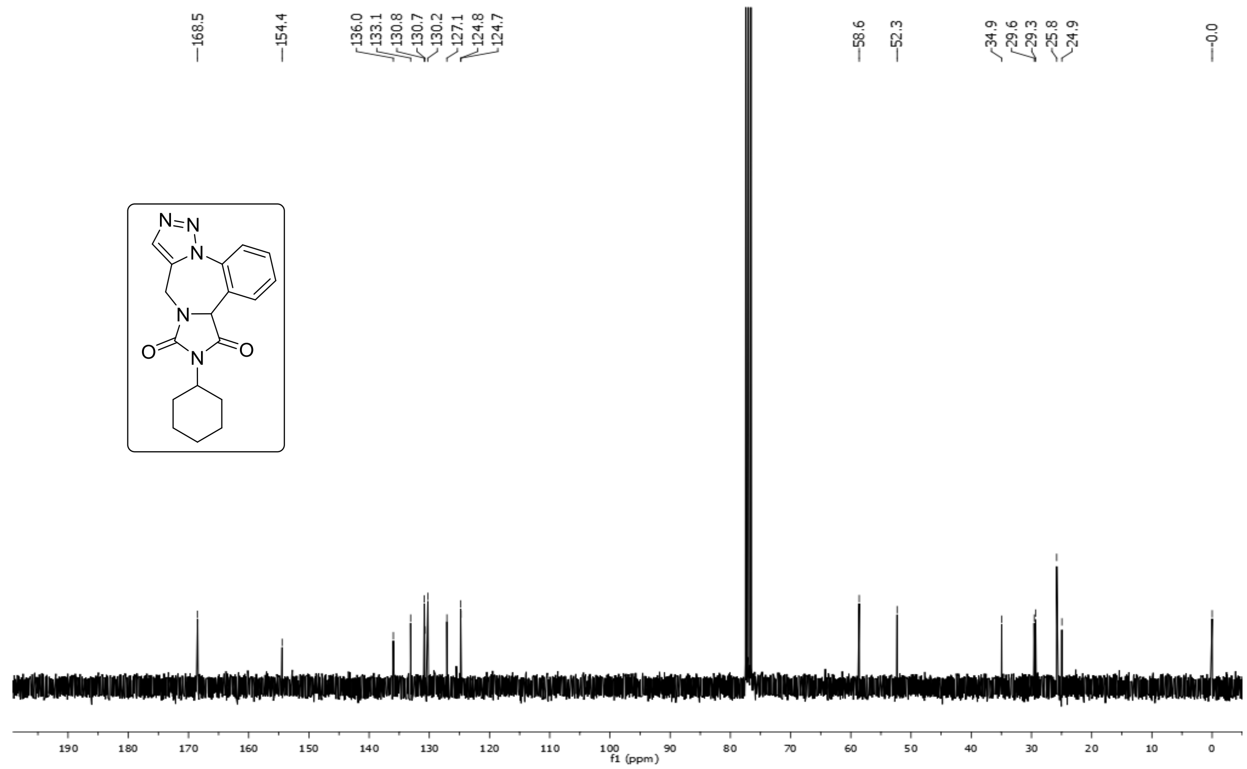

10c (^{1}H NMR, 300 MHz)

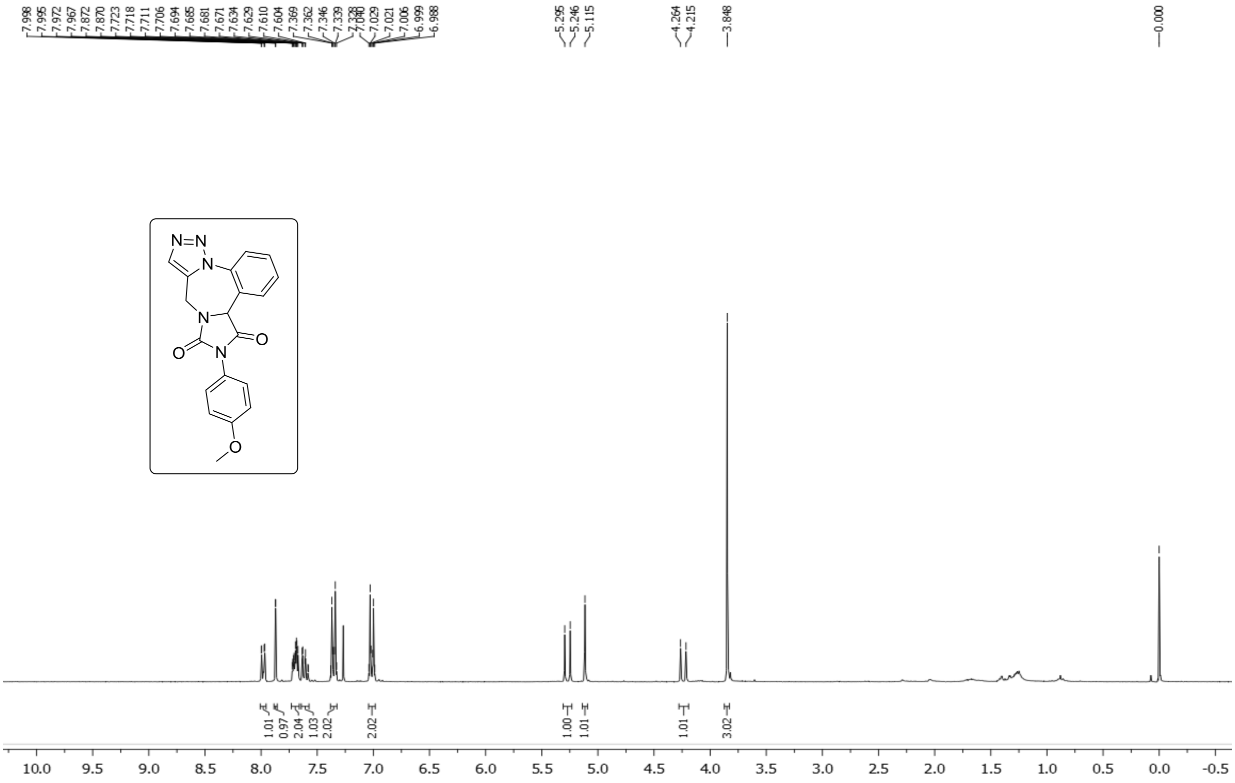

10c (^{13}C NMR, 75 MHz)

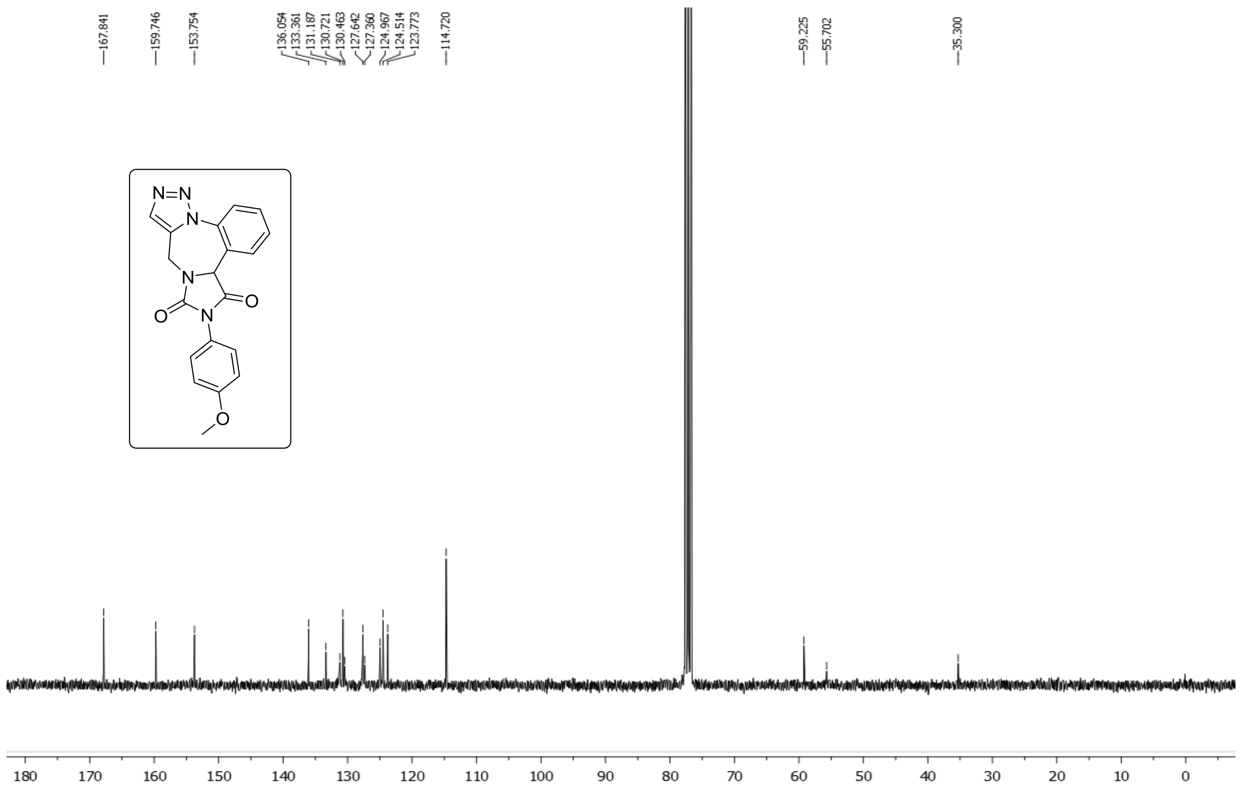

Crystal structure determination of 10a

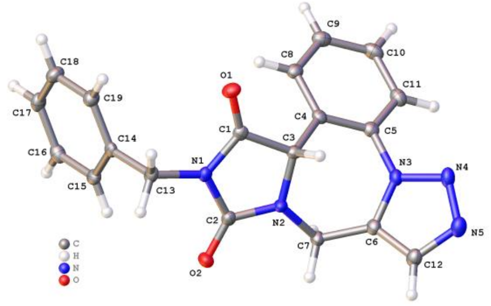

Single crystals of **10a** were grown from methanol. A suitable crystal was selected and X-ray diffraction data were collected at 99.99(10) K on an Agilent SuperNova diffractometer with Eos CCD detector using Mo K_α radiation. Data frames were processed (unit cell determination, intensity data integration, correction for Lorentz and polarization effects, and empirical absorption correction) using CrysAlis PRO [6]. Using Olex2 [7], the structure was solved with the ShelXS [8] structure solution program using Direct Methods and refined with the ShelXL [9] refinement package using full-matrix least-squares minimisation. All H atoms were placed in idealized positions and refined in the riding mode, with U_{iso}(H) values assigned as 1.2U_{eq} of the parent atoms, and with C—H distances of 0.95 (aromatic), 0.99 (CH_2) and 1.00 Å (CH). Due to the presence of only C, N, O and H atoms, the determination of the absolute configuration by refinement of the Flack parameter was not possible. Crystal data, data collection and structure refinement details are summarized in Table S1. Crystal structure data have been deposited at the Cambridge Crystallographic Data Centre, and allocated the deposition number CCDC 1546593.

Table S1: Crystal data and structure refinement for 10a.

Empirical formula	$C_{19}H_{15}N_5O_2$
Formula weight	345.36
Temperature/K	99.99(10)
Crystal system	monoclinic
Space group	$P2_1$
a/Å	10.2729(5)

b/Å	7.9593(3)
c/Å	10.5905(5)
α/°	90
β/°	113.069(6)
γ/°	90
Volume/$Å^3$	796.69(7)
Z	2
ρ_{calc}g/cm^3	1.440
μ/mm^{-1}	0.098
F(000)	360.0
Crystal size/mm^3	0.3 × 0.2 × 0.2
Radiation	Mo K_α (λ = 0.71073 Å)
2Θ range for data collection/°	6.61 to 52.744
Index ranges	-12 ≤ h ≤ 12, -9 ≤ k ≤ 9, -13 ≤ l ≤ 13
Reflections collected	16374
Independent reflections	3261 [R_{int} = 0.0353, R_{sigma} = 0.0217]
Data/restraints/parameters	3261/1/235
Goodness-of-fit on F^2	1.142
Final R indexes [I>=2σ (I)]	R_1 = 0.0290, wR_2 = 0.0761
Final R indexes [all data]	R_1 = 0.0297, wR_2 = 0.0768
Largest diff. peak/hole / e $Å^{-3}$	0.23/-0.18
Flack parameter	0.6(4)

References

1. Hashmi, A. S. K.; Haufe, P.; Nass, A. R.; Bats, J. W. *Adv. Synth. Catal.* **2004**, *346*, 421-424.
2. Pelkey, E. T.; Gribble, G. W. *Tetrahedron Lett.* **1997**, *38*, 5603-5606.
3. Guggenheim, K. G.; Toru, H. *Org. Lett.* **2012**, *14*, 3732-3735.
4. Vidyacharan, S.; Murugan, A.; Sharada, D. S. *J. Org. Chem.* **2016**, *81*, 2837-2848.
5. Hu, D.; Grice, P.; Ley, S. V. *J. Org. Chem.* **2012**, *77*, 5198.
6. Dolomanov, O.V.; Bourhis, L.J.; Gildea, R.J.; Howard, J. A. K.; Puschmann, H. *J. Appl. Cryst.* **2009**, *42*, 339-341.
7. Agilent, *CrysAlis PRO*. **2011**, Agilent Technologies, Yarnton, England.
8. Sheldrick, G.M. *Acta Cryst.* **2008**, *A64*, 112-122.
9. Sheldrick, G.M. *Acta Cryst.* **2015**, *C71*, 3-8.
